# CircSLC7A2 protects against osteoarthritis through inhibition of the miR‐4498/TIMP3 axis

**DOI:** 10.1111/cpr.13047

**Published:** 2021-05-07

**Authors:** Weiyu Ni, Chao Jiang, Yizheng Wu, Haitao Zhang, Lili Wang, Jasper H.N. Yik, Dominik R. Haudenschild, Shunwu Fan, Shuying Shen, Ziang Hu

**Affiliations:** ^1^ Department of Orthopaedic Surgery Sir Run Run Shaw Hospital Zhejiang University School of Medicine Hangzhou China; ^2^ Key Laboratory of Musculoskeletal System Degeneration and Regeneration Translational Research of Zhejiang Province Hangzhou China; ^3^ School of Statistics and Mathematics Zhejiang Gongshang University Hangzhou PR China; ^4^ Ellison Musculoskeletal Research Center Department of Orthopaedic Surgery University of California System Davis CA USA

**Keywords:** circSLC7A2, degenerative disease, extracellular matrix, miR‐4498, osteoarthritis, TIMP3

## Abstract

**Objectives:**

Circular RNAs (circRNAs) are noncoding RNAs that compete against other endogenous RNA species, such as microRNAs, and have been implicated in many diseases. In this study, we investigated the role of a new circRNA (circSLC7A2) in osteoarthritis (OA).

**Materials and Methods:**

The relative expression of circSLC7A2 was significantly lower in OA tissues than it was in matched controls, as shown by real‐time quantitative polymerase chain reaction (RT‐qPCR). Western blotting, RT‐qPCR and immunofluorescence experiments were employed to evaluate the roles of circSLC7A2, miR‐4498 and TIMP3. The in vivo role and mechanism of circSLC7A2 were also conformed in a mouse model.

**Results:**

circSLC7A2 was decreased in OA model and the circularization of circSLC7A2 was regulated by FUS. Loss of circSLC7A2 reduced the sponge of miR‐4498 and further inhibited the expression of TIMP3, subsequently leading to an inflammatory response. We further determined that miR‐4498 inhibitor reversed circSLC7A2‐knockdown‐induced OA phenotypes. Intra‐articular injection of circSLC7A2 alleviated in vivo OA progression in a mouse model of anterior cruciate ligament transection (ACLT).

**Conclusions:**

The circSLC7A2/miR‐4498/TIMP3 axis of chondrocytes catabolism and anabolism plays a critical role in OA development. Our results suggest that circSLC7A2 may serve as a new therapeutic target for osteoarthritis.

## Graphical abstract

The expression of the novel circular RNA, circSLC7A2, is important for maintaining ECM homeostasis and protecting cartilage against OA progression. CircSLC7A2 is regulated by FUS and functions through miR‐4498/TIMP3 axis, which is effective and comprehensive in alleviating inflammation, inhibiting catabolic enzymes, introducing anabolic enzymes and preventing cell apoptosis that resulted in maintaining cartilage ECM, thus preventing or delaying OA progression.

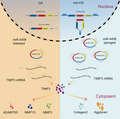

**Abbreviations**
AAV: adeno‐associated virus
ACLT: anterior cruciate ligament transection
ADAMTS5: a disintegrin and metalloproteinase with thrombospondin motifs‐5
BSA: bovine serum albumin
ceRNA: competing endogenous RNA
circRNA: circular RNA
DMEM: Dulbecco's modified Eagle's medium
ECM: extracellular matrix
EDTA: ethylenediaminetetraacetic acid
FBS: foetal bovine serum
FISH: fluorescence in situ hybridization
gDNA: Genomic DNA
HC: human cartilage
HRP: horseradish peroxidase
IF: immunofluorescence
IHC: immunohistochemistry
miRNA: microRNA
MMP13: matrix metalloproteinase 13
MMP3: matrix metalloproteinase 3
OA: osteoarthritis
PBS: phosphate‐buffered saline
PGs: proteoglycans
pre‐mRNA: mRNA precursors
RBP: RNA‐binding protein
RIP: RNA immunoprecipitation
RT‐qPCR: quantitative real‐time polymerase chain reaction
SLC7A2: solute carrier family 7 member 2
TAE: Tris‐acetate‐EDTA
TBS: Tris‐HCl buffer
TIMP3: tissue inhibitor of metalloproteinase 3

## 1 INTRODUCTION

Osteoarthritis (OA) is a common degenerative joint disease that affects more than 10% of the adult population.^1^, ^2^ The development of OA is influenced by a variety of factors, including ageing, genetic make‐up, mechanical over loading and multiple inflammatory/catabolic pathways, leading to synovial inflammation, cartilage degradation and subchondral bone remodelling.^3^, ^4^, ^5^, ^6^ The extracellular matrix (ECM) in cartilage tissues is the main component responsible for load absorption in joints.^7^ ECM accumulation is promoted by the expression of collagen II and aggrecan. In contrast, the degradation of ECM is enhanced by the expression of matrix metalloproteinases (MMPs), such as MMP3 and MMP13, and a disintegrin and metalloproteinase with thrombospondin motif (ADAMTS) proteins, such as ADAMTS4 and ADAMTS5.^8^, ^9^ Although the uncontrolled proteolytic degradation of the ECM in cartilage is an initiating factor for OA,^10^ the exact mechanism of OA pathogenesis is still unknown and there is currently no effective clinical therapy to cure or prevent OA.

Recently, biologics have become a thriving therapeutic approach to treat diseases such as arthritis.^11^ Tumour necrosis factor antagonists (etanercept, infliximab and adalimumab), an interleukin‐1 receptor antagonist (anakinra) and a monoclonal antibody against CD‐20‐positive B cells (rituximab) are among the widely used biologics that have demonstrated clinical efficacy in treating rheumatoid arthritis.^12^, ^13^ Thus, biologics hold great promise for arthritis treatment. However, few biologics have been tested for the treatment of OA.

Biologics, such as circRNAs, have been reported to play critical roles in degenerative diseases by regulating ECM metabolism.^8^, ^14^ Circular RNAs (circRNAs) are noncoding RNAs characterized by a covalent loop configuration without a polyadenylated tail and have been found to have diverse but important physiological functions.^15^, ^16^ CircRNAs are derived from mRNA precursors (pre‐mRNAs), which are then circularized by back‐splicing between a 5’ and 3’ splice site.^17^, ^18^ In recent years, circRNAs have become the focus of many studies. For instance, circHIPK3 is a key autophagy regulator of lung cancer,^19^ circHIPK2 promotes astrocyte activation via the regulation of autophagy and endoplasmic reticulum stress,^20^ and circFNTA activates Kirsten rat sarcoma viral oncogene signalling to promote bladder cancer progression.^21^ CircRNAs exert their functions via several mechanisms to pleiotropically regulate gene expression. One such mechanism is to compete against endogenous microRNAs (miRNAs).^22^ Complementary sequences of circRNAs promote the binding and sequestering of endogenous miRNAs to inhibit their functions.^23^, ^24^, ^25^

Based on our previous RNA sequencing (RNA‐seq) analyses of differentially expressed circRNAs in human OA cartilage, the 25 most differentially expressed circRNAs were identified, of which 14 of them were significantly downregulated.^26^ One of the downregulated circRNAs (hsa_circ_0005805) likely plays an important role in OA progression. According to circBank, hsa_circ_0005805 is also named hsa_circSLC7A2_010, hereafter referred to as circSLC7A2. CircSLC7A2 is a circularized mRNA product of the solute carrier family 7 member 2 (SLC7A2) gene, which is an inducible transporter of the semi‐essential amino acid L‐arginine. It has been reported to exert functions in many tissues, such as macrophages, colonic epithelial cells and mouse cells.^27^, ^28^, ^29^ In this study, the role of circSLC7A2 in regulating ECM metabolism was investigated and its function and targets were characterized using in vitro and in vivo models of OA.

## 2 EXPERIMENTAL PROCEDURES

Detailed experimental procedures are described in the supplementary materials and methods.

## 3 RESULTS

### 3.1 CircSLC7A2 is downregulated in osteoarthritic cartilage

**FIGURE 1:**
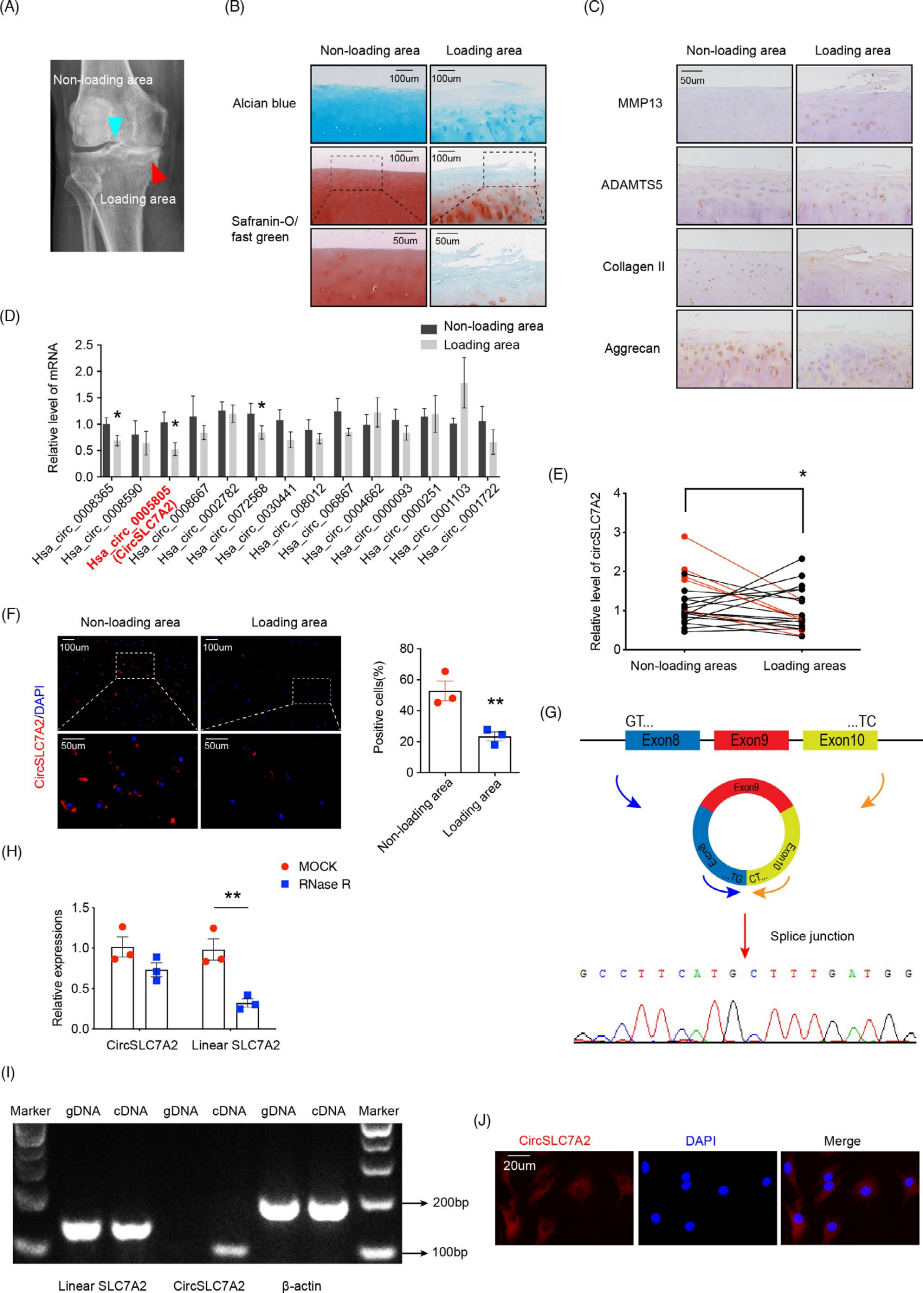
(A) X‐ray of human knee joint with OA. (B) Safranin O/fast green and Alcian blue staining of femoral condyles (scale bar = 50‐100μm). (C) MMP13, ADAMTS5, aggrecan and type II collagen expression in knee joints were detected by IHC (scale bar = 50 μm). Data are representative images of similar results obtained from three different donors or three independent experiments. (D) Relative expression of 14 circRNAs in loading and non‐loading areas (n = 3). The expression levels of the 14 candidates in loading areas were normalized to non‐loading areas. (*P < .05, comparative t test) (E) Relative expression of circSLC7A2 in loading and non‐loading areas (n = 28 donors), circSLC7A2 expressions in loading areas/non‐loading areas >2 were shown in red (*P < .05, comparative t test). (F) Detection of circSLC7A2 in cartilage by FISH. Nuclei were stained with DAPI (scale bar = 50‐100μm, *P < .05, Student's t test). (G) Schematic showing the circularization of circSLC7A2 via splicing of exons 8 and 10. The presence of circSLC7A2 was validated by Sanger sequencing. (H) The relative expression of circSLC7A2 and its mRNA precursor (linear SLC7A2) in HC cells after RNase R treatment. The expression of circSLC7A2 and linear SLC7A2 in mock‐treated samples was arbitrarily set to 1. Data are presented as the mean ± SEM from three independent experiments. (I) Validation of circSLC7A2 expression in HC cells by nucleic acid electrophoresis. Divergent primers amplified circSLC7A2 in cDNA but not genomic DNA (gDNA). Primers for β‐actin were used as a negative control. (J) Predominately cytoplasmic expression of circSLC7A2 in HC cells detected by FISH. Nuclei were stained with DAPI (scale bar = 20 μm). Data are representative images of similar results obtained from three different donors (A, B, C, F, I and J) or presented as the mean ± SEM from three independent experiments (H). (*P < .05, **P < .01 vs control or as indicated by the Student's t test)

RNA‐seq analyses in our previous study revealed a series of differentially expressed circRNAs between OA (age‐related) and non‐OA cartilage samples (Figure S1A). A total of 14 significantly downregulated circRNAs were reported.^26^ OA is typically found in loading areas rather than non‐loading areas because of uneven weight bearing(Figure 1A).^30^, ^31^ In this study, differentially expressed circRNAs in human osteoarthritic cartilage from loading areas were identified. Normal cartilage from non‐loading areas was used as individual controls to minimize confounding effects in the analysis due to donor variations. We first performed histological analyses to confirm the OA status of the paired loading and non‐loading areas from the patients. Safranin O and Alcian blue staining showed that proteoglycan (PG) levels were reduced in arthritic loading areas compared to healthy non‐loading areas (Figure 1B). Immunohistochemistry (IHC) also revealed higher levels of the degradative enzymes MMP13 and ADAMTS5 but reduced expression levels of the matrix proteins collagen II and aggrecan in arthritic loading areas (Figure 1C). These results are consistent with the arthritic phenotype expected in the OA cartilage. Next, we used real‐time quantitative polymerase chain reaction (RT‐qPCR) to determine the relative expression levels of these 14 circRNAs in clinical cartilage specimens isolated from paired loading and non‐loading areas (n = 3) (Figure 1D). As a result, hsa_circ_0005805 (circSLC7A2) was found to be the most significantly decreased in the loading area. To further identify the expression of circSLC7A2 in loading and non‐loading areas, we collected cartilage tissues in loading areas from 28 donors and selected paired samples from non‐loading areas as negative controls (NCs). CircSLC7A2 expression was normalized to that in the negative control samples, and the results showed that circSLC7A2 expression was significantly lower in OA cartilage (loading areas) than in non‐OA cartilage (non‐loading areas) (Figure 1E). The reduced expression of circSLC7A2 in OA cartilage was further confirmed by FISH analysis (Figure 1F). Through these experiments, we identified that circSLC7A2 expression was reduced under osteoarthritic conditions.

### 3.2 Confirmation of circSLC7A2 expression in chondrocytes

By comparing the circSLC7A2 sequence and its parental gene according to the circBase database and NCBI, we investigated whether circSLC7A2 was looped and contained exons 8‐10 of its parental gene. To distinguish between SLC7A2 and circSLC7A2, circSLC7A2‐specific primers were designed such that the PCR product must span the joining site between the 3’ and 5’ ends of circSLC7A2, which does not exist in the linear RNA. Sanger sequencing of the circSLC7A2 PCR product confirmed that circSLC7A2 was back‐spliced from the 5’ splice site of exon 8 to the 3’ splice site of exon 10 (Figure 1G). However, genomic rearrangements can also be back‐spliced. Thus, several steps were taken to rule out this possibility. First, circSLC7A2 was compared to its mRNA precursor (NM_001008539); circSLC7A2 was found to be more resistant to digestion by RNase R, which preferentially degrades linear RNA species (Figure 1H). Second, genomic DNA (gDNA) and total RNA were extracted separately from human chondrocytes. Total RNA contained both the linear mRNA precursor (SLC7A2) and the mature circSLC7A2. cDNA was then generated and subjected to PCR using different primer pairs that can distinguish the linear and circular forms of circSLC7A2. The expected PCR products had lengths of 140 and 100 bp, respectively. Electrophoresis detected the expected 140 bp bands from both gDNA and cDNA. However, the 100 bp band was visible only in the cDNA samples, indicating the presence of circSLC7A2. (Figure 1I). Taken together, these data confirmed circSLC7A2 expression in human chondrocytes. Finally, FISH analysis showed that circSLC7A2 expression was predominantly cytoplasmic in human chondrocytes (Figure 1J).

### 3.3 CircSLC7A2 is involved in the regulation of cartilage homeostasis and chondrocyte apoptosis

**FIGURE 2:**
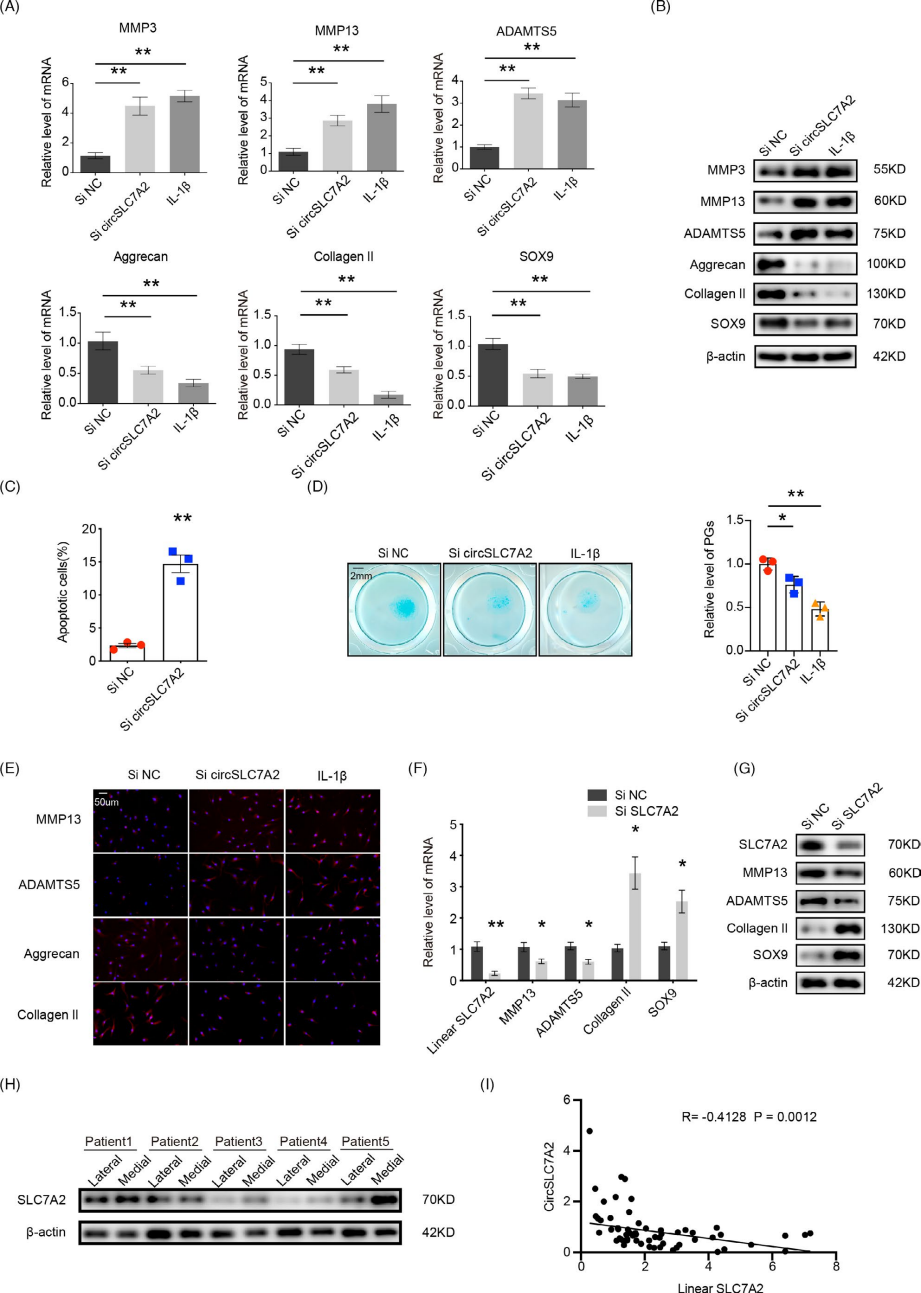
(A & B) MMP3, MMP13, ADAMTS5, aggrecan, collagen II and SOX9 RNA levels in HC cells transfected with si‐circSLC7A2 or treated with IL‐1β; and Western blotting. (C) Apoptosis flow cytometry detection is shown after Annexin V‐FITC/propidium iodide (PI) dual staining. (D) Representative images of HC cells stained with Alcian blue, scale bar =2 mm. Relative values of proteoglycans (PGs) were downregulated in HC cells transfected with si‐circSLC7A2 or treated with IL‐1β. (E) IF for MMP13, ADAMTS5, aggrecan and collagen II expression detection in HC cells. Nuclei were stained with DAPI (scale bar=50 μm). The transfection of circSLC7A2 and negative control in HC cells. (F & G) MMP13, ADAMTS5, collagen II and SOX9 RNA and protein levels in HC cells transfected with si SLC7A2. (H) WB for SLC7A2 in loading and non‐loading areas in different people (n = 5). (I) Pearson correlation analysis showed a significant positive correlation of SLC7A2 vs circSLC7A2 normalized to pre‐SLC7A2. (Pearson's r = −0.4128; *P* = .0012, n = 59). Data are representative images of similar results obtained from three different donors (B, D, E, G and H) or presented as the mean ± SEM from three independent experiments (A, C, F and I). (*P < .05, **P < .01 vs control or as indicated by the Student's t test)

Next, we investigated the role of circSLC7A2 in cartilage. WB and RT‐qPCR demonstrated the association of circSLC7A2 overexpression with the protein expression of matrix‐degrading and synthesizing components (Figure S1B‐D). Three siRNAs against circSLC7A2 were designed to specifically target the junction site, but not linear SLC7A2 mRNA. The ability of these siRNAs to degrade circSLC7A2 in transfected chondrocytes was assessed by RT‐qPCR. The results showed that si‐circSLC7A2‐2 was the most effective at knocking down circSLC7A2 expression by more than 80% (Figure S2A). In contrast, the levels of the linear SLC7A2 mRNA were not affected (Figure S2B). Therefore, si‐circSLC7A2‐2 was used in subsequent experiments. First, the effects of silencing circSLC7A2 on the expression of genes important for cartilage ECM homeostasis were investigated by treating human cartilage (HC) cells with IL‐1β as a positive control. The knockdown of circSLC7A2 in chondrocytes resulted in an increased protein expression of catabolic enzymes MMP3, MMP13 and ADAMTS5, but a decreased the expression of anabolic factors collagen II, aggrecan and Sox9 (Figure 2A). In addition to gene expression, similar results were obtained by Western blotting (Figure 2B) and immunofluorescence (IF) staining (Figure 2E, Figure S2F) in HC cells. The results also showed the mRNA and protein levels of catabolic and anabolic enzymes in SW1353 cells (Figure S2C and D). Next, we investigated the role of circSLC7A2 in chondrocyte apoptosis using flow cytometry. The results showed that circSLC7A2 knockdown caused an increase in apoptotic chondrocytes by approximately 10% (Figure 2C), and SW1353 cells (Figure S2E). In addition, micro‐mass culture followed by Alcian blue staining showed a decreased proteoglycan distribution after circSLC7A2 knockdown (Figure 2D). To distinguish between the function of circular circSLC7A2 and linear SLC7A2 transcripts, we used siRNAs specific for linear SLC7A2 and transfected them into HC cells. The RT‐qPCR and Western blotting results showed that the knockdown of SLC7A2 increased the levels of anabolic enzymes and decreased those of catabolic enzymes (Figure 2F and G). Western blotting also identified higher levels of SLC7A2 expression in representative loading areas compared to non‐loading areas (Figure 2H). To analyse the correlation between linear SLC7A2 and circSLC7A2, Pearson correlation analysis was performed. The results showed a significant negative correlation between linear SLC7A2 and circSLC7A2 (normalized to pre‐SLC7A2) among HC cells extracted from cartilage from different people (Figure 2I). Collectively, these results indicate that circSLC7A2 plays a critical role in maintaining the anabolic state in HC cells, and that linear SLC7A2 plays the opposite role in OA progression.

### 3.4 RNA‐binding protein (RBP) FUS promotes circSLC7A2 production by binding to the flanked intron regions of circSLC7A2

**FIGURE 3:**
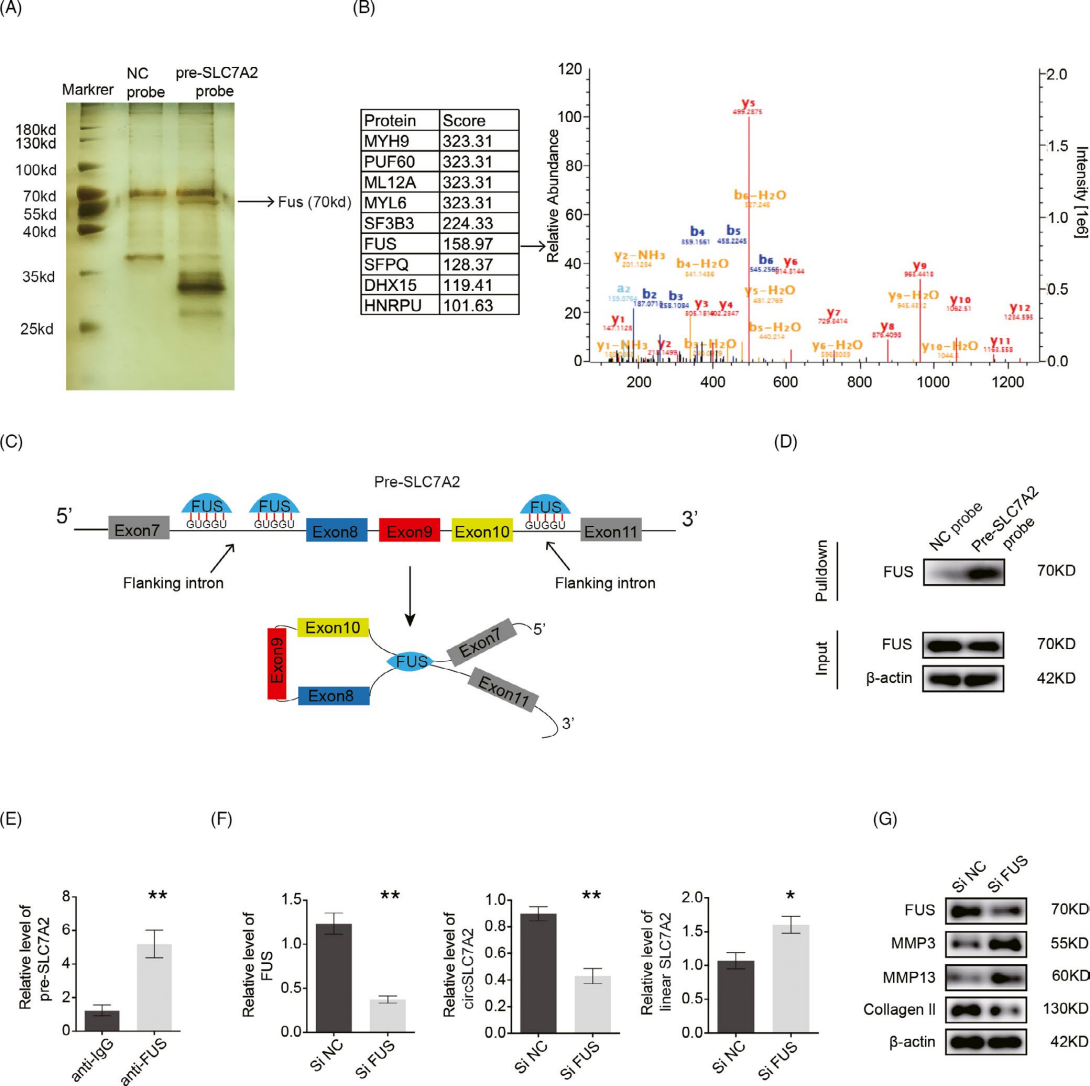
(A) Silver staining of the pre‐SLC7A2‐protein complex pulled down by pre‐SLC7A2 probe. (B) The ten proteins with the highest score by mass spectrometry analysis and best peptide spectrum of FUS. (C) Schematic model of FUS‐mediated circSLC7A2 produced via 3 binding sites (interacting with ‘GUGGU’ motifs) on the flanked intron regions of circ SLC7A2‐forming exons. (D) WB analysis of FUS after pull‐down assay. (E) RIP assays showing the association of FUS with pre‐SLC7A2 in SW1353. (F) HC cells were transfected with si‐NC or si‐FUS. FUS, circSLC7A2 and SLC7A2 expression were determined by RT‐qPCR. (G) FUS, MMP3, MMP13 and collagen II protein levels in HC cells transfected with si‐NC and si‐FUS. Data are representative images of similar results obtained from three different donors (D and G) or presented as the mean ± SEM from three independent experiments (E and F). (*P < .05, **P < .01 vs control or as indicated the Student's t test)

Since linear SLC7A2 plays an opposing role to that of circSLC7A2 in regulating the ECM and is negatively correlated with circSLC7A2, the formation of linear SLC7A2 and circSLC7A2 may be an important factor in OA progression. Derived from canonical splice sites, circRNAs are generally cyclized via the following three classical mechanisms: (1) intron pairing; (2) exon skipping and intron lariat formation; and (3) RBP‐mediated events.^32^ As there are many protein‐binding sites in the flanking introns of circSLC7A2, we considered that RBPs might promote its circularization. First, we performed RNA pull‐down assays with a probe targeting the circSLC7A2 flanking introns within pre‐SLC7A2 followed by silver staining and mass spectrometry. (Figure 3A and B). We found that numerous proteins could be pulled down by pre‐SLC7A2. Mass spectrometry analysis was used to further screen the pre‐SLC7A2 pull‐down products and showed the 10 proteins with the highest scores among the total 300 proteins. A previous study revealed that FUS participates in several RNA biosynthetic processes.^33^ Therefore, we focussed on FUS, an RBP trans‐factor that has been reported to regulate circRNA generation primarily through two GUGGU‐binding motifs with high scores.^34^ Since the regulation of circRNA back‐splicing normally involves the introns flanking circularized exons, we found two GUGGU sequences at nt 358 and 409 proximal to the back‐splicing sites (exon 8) and one sequence at nt 499 proximal to the back‐splicing sites (exon 10) (Figure 3C). Western blot analysis confirmed the pull down of the FUS protein by the pre‐SLC7A2 probe (Figure 3D). Furthermore, an RNA immunoprecipitation (RIP) assay with an anti‐FUS or control IgG antibody confirmed the direct interaction between FUS and pre‐SLC7A2 (Figure 3E). Next, we transfected si‐FUS into HC cells and found that after FUS knockdown, circSLC7A2 was downregulated, while linear SLC7A2 was upregulated, by RT‐qPCR (Figure 3F). Further exploring the function of FUS in chondrocytes, HC cells transfected with si‐FUS were found to upregulate the degradative enzymes MMP3 and MMP13 and downregulate the matrix protein collagen II (Figure 3G), in accordance with the above results. Taken together, these findings indicate that circSLC7A2 circularization can be mediated by FUS, which plays an important role in ECM metabolism.

### 3.5 CircSLC7A2 functions in OA by targeting miRNA expression

**FIGURE 4:**
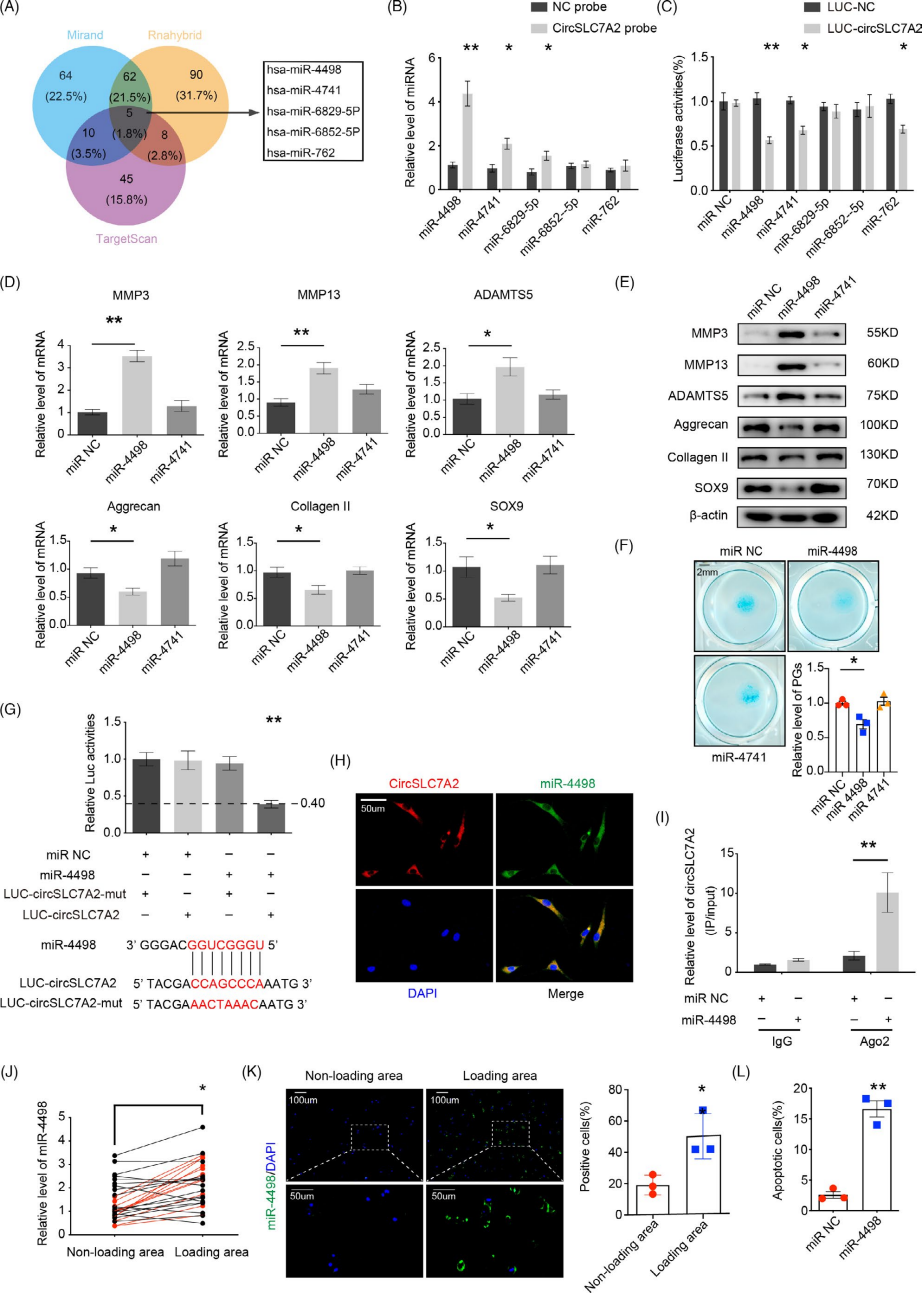
(A) Schematic illustration of the 5 overlapping candidate miRNAs predicted to be targets of circSLC7A2, by the bioinformatics analysis of miRanda, RNAhybrid and TargetScan. (B) SW1353 cells were subjected to an RNA pull‐down assay with circSLC7A2 or negative control probe and tested by RT‐qPCR analysis. The interaction of the 5 candidates with circSLC7A2 was normalized to the negative control probe. (C) HEK‐293 cells were transfected with miR‐negative control (NC) or five predicted miRNAs and then transfected with luciferase constructs of wild‐type circSLC7A2 or negative control. Luciferase reporter assay found that miR‐4498 exclusively decreased the luciferase activity of wild‐type reporter plasmids. n = 6; *P < .05, **P < .01. (D & E) MMP3, MMP13, ADAMTS5, aggrecan, collagen II and SOX9 protein levels in HC cells transfected with negative control, miR‐4498, and miR‐4741 as well as the RT‐qPCR results. (F) Representative images of HC cells stained with Alcian blue that transfected with negative control, miR‐4498, and miR‐4741, scale bar =2 mm. (G) HEK‐293 cells were transfected with miR‐4498 or negative control and then transfected with luciferase constructs of wild‐type circSLC7A2 or mutated circSLC7A2. (H) miR‐4498 was detected in HC cells by FISH and its specific probe was labelled with Alexa Fluor 488, while circSLC7A2 probes were labelled with Alexa Fluor 555 and nuclei were stained with DAPI (scale bar = 50 μm). (I) AGO2 RIP assay was performed to detect the circSLC7A2 levels in SW1353 cells transfected with miR‐4498 or negative control. (J & K) miR‐4498 expression was higher in cartilage of human loading areas than it was in non‐loading areas, as determined by RT‐qPCR (J), n = 28, miR‐4498 expressions in loading areas/non‐loading areas < 0.5 were shown in red (*P < .05, comparative t test); a similar finding was shown by FISH (*P < .05, Student's t test). (K). Nuclei were stained with DAPI (scale bar = 50‐100μm, *P < .05, Student's t test). (L) Apoptosis flow cytometry detection is shown after Annexin V‐FITC/propidium iodide (PI). The transfection of miR‐4498 and negative control in HC cells. Data are representative images of similar results obtained from three different donors (E, F, H, and K) or presented as the mean ± SEM from three independent experiments (B, C, D, G, I and L). (*P < .05, **P < .01 vs control or as indicated by the Student's t test)

CircRNAs can act like sponges, binding and inducing the degradation of endogenous miRNAs, thereby inhibiting their mRNA‐targeting function. Therefore, we explored whether circSLC7A2 acts as a miRNA sponge in chondrocytes. A total of five candidate miRNAs were predicted to be targets of circSLC7A2 based on overlapping bioinformatics analysis from three databases (miRanda, RNAhybrid and TargetScan) (Figure 4A). RNA pull‐down assays and subsequent RT‐qPCR showed the relative levels of these five endogenous miRNAs in chondrocytes in the presence or absence of the circSLC7A2 probe. The results showed that the relative levels of miR‐4498, miR‐4741 and miR‐6829‐5p were significantly upregulated, with miR‐4498 being the most affected (Figure 4B). The binding of reporter plasmids to the miRNAs affects luciferase activity, and the luciferase assay results demonstrated that among these miRNAs, miR‐4498 had the most significant change in luciferase intensity (Figure 4C). Taken together, these findings indicate that circSLC7A2 acts as a sponge to target miR‐4498 or miR‐4741. To investigate the roles of the two miRNA targets of circSLC7A2 in chondrocytes, we transfected miR‐4498 and miR‐4741 mimics into HC cells and then examined the expression of catabolic and anabolic factors by Western blot analysis and RT‐qPCR. The results demonstrated that between the two miRNAs, miR‐4498 had more significant effects on increasing the protein and mRNA levels of the matrix‐degrading enzymes MMP3, MMP13 and ADAMTS5 while decreasing the expression of the anabolic factors collagen II, aggrecan and SOX9 (Figure 4D and E). Additionally, miR‐4498 decreased the proteoglycan distribution, as confirmed by Alcian blue staining (Figure 4F). These data indicate that miR‐4498 is most likely the target of circSLC7A2.

### 3.6 CircSLC7A2 acts as a sponge for miR‐4498

Next, we identified the potential binding sequence of circSLC7A2 and miR‐4498, using bioinformatics analysis of the CircInteractome database (Figure 4G). We constructed reporter plasmids containing the circSLC7A2 sequence and mutated reporter plasmids. Our results showed that the luciferase activity of the wild‐type reporter was 60% lower than that of the mutant reporter. To further investigate circSLC7A2 sponging of miR‐4498, we conducted FISH experiments, which confirmed the colocalization of circSLC7A2 and miR‐4498 in HC cells (Figure 4H). Since miRNAs bind to circRNAs via the AGO2 complex, we conducted RIP assays, which showed that more endogenous circSLC7A2 was pulled down from SW1353 cells by anti‐AGO2 antibodies than by IgG. In SW1353 cells transfected with miR‐4498 and the negative control, we observed a ten‐fold increase in circSLC7A2 associated with AGO2 by overexpressing miR‐4498 (Figure 4I). Furthermore, RNA FISH in tissues and RT‐qPCR experiments confirmed that miR‐4498 was abundant in loading areas (Figure 4J and K), and miR‐4498 was found to be increased in SW1353 cells after induction with IL‐1β (Figure S3A). To identify the function of miR‐4498, HC cells were transfected with miR‐4498 mimics; the transfection efficiency of miR‐4498 mimics increased the levels of the miRNA by 230‐fold, as determined by RT‐qPCR (Figure S3B). The overexpression of miR‐4498 significantly increased the percentage of apoptotic HC cells (Figure 4L). Additionally, the influence of miR‐4498 was found to influence protein and mRNA expression, as well as cell viability, in SW1353 cells (Figure S3C‐F). These results indicate that circSLC7A2 sponges miR‐4498.

### 3.7 Inhibiting miR‐4498 reverses circSLC7A2‐knockdown‐induced OA and IL‐1β‐induced inflammation

**FIGURE 5:**
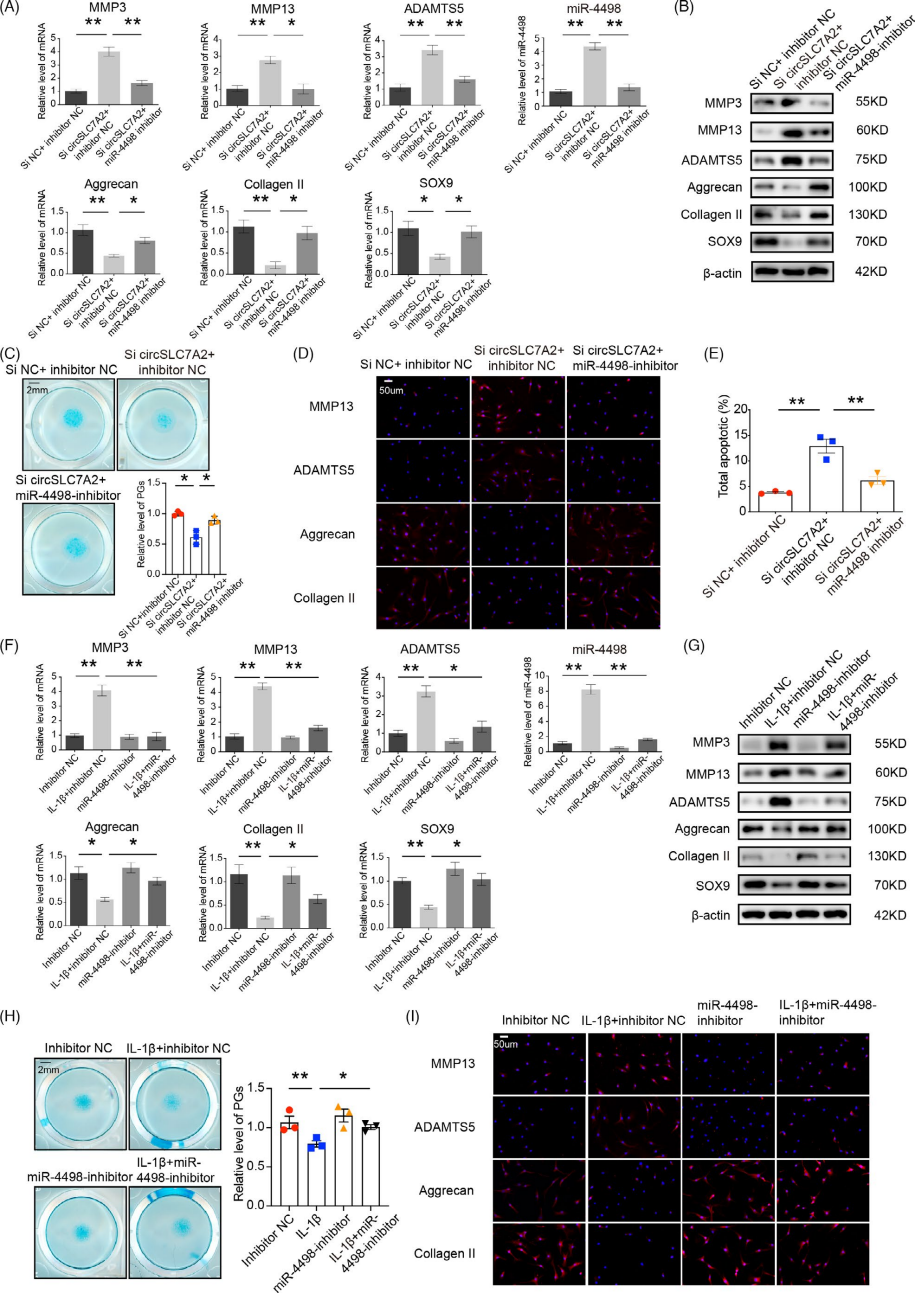
(A & B) MMP3, MMP13, ADAMTS5, aggrecan, collagen II and SOX9 mRNA expressions, and protein synthesis in HC cells that were co‐transfected with si‐circSLC7A2 and miR‐4498‐inhibitor. (C) Representative images of HC cells stained with Alcian blue and co‐transfected with si‐circSLC7A2 and miR‐4498‐inhibitor, scale bar = 2 mm. (D) MMP13, ADAMTS5, Aggrecan and Collagen II expression in HC cells that co‐transfected with si‐circSLC7A2 and miR‐4498‐inhibitor was detected by IF analysis. Nuclei were stained with DAPI (scale bar = 50μm). (E) Apoptosis flow cytometry detection is shown after Annexin V‐FITC/propidium iodide (PI). The co‐transfection of si‐circSLC7A2 with miR‐4498 or negative control in HC cells. (F & G) HC cells were transfected with miR‐4498‐inhibitor or negative control, and then exposed to IL‐1β. WB and RT‐qPCR for MMP3, MMP13, ADAMTS5, aggrecan, collagen II and SOX9 detection. (H) Representative images of HC cells stained with Alcian blue and transfected with miR‐4498‐inhibitor or negative control, and then exposed to IL‐1β, scale bar =2 mm. (I) IF analysis was used to determine the expression of MMP13, ADAMTS5, aggrecan and collagen II in HC cells treated with miR‐4498‐inhibitor or negative control, and then exposed to IL‐1β. Nuclei were stained with DAPI (scale bar = 50μm). Data are representative images of similar results obtained from three different donors (B, C, D, G, H and I) or presented as the mean ± SEM from three independent experiments (A, E and F). (*P < .05, **P < .01 vs control or as indicated by the Student's t test)

We co‐transfected si‐circSLC7A2 and a miR‐4498 inhibitor into HC and SW1353 cells to investigate whether inhibiting miR‐4498 could rescue the effects of circSLC7A2 loss. The knockdown of circSLC7A2 expression increased the miR‐4498 levels and MMP3, MMP13 and ADAMTS5 mRNA and protein levels but decreased the levels of aggrecan, collagen II and SOX9. As expected, these effects were markedly rescued by treatment with the miR‐4498 inhibitor (Figure 5A and B). Alcian blue staining also showed the rescue of the proteoglycan distribution when miR‐4498 was inhibited (Figure 5C), and the IF assay in HC cells confirmed these results noted above (Figure 5D, Figure S4D). Moreover, HC cells co‐transfected with a miR‐4498 inhibitor and si‐circSLC7A2 showed a rescue effect in terms of apoptosis (Figure 5E). Similar results were also observed in SW1353 cells (Figure S4A‐C). Since IL‐1β simulates an osteoarthritic condition, we investigated whether the miR‐4498‐inhibitor could rescue the influence of IL‐1β. The miR‐4498 inhibitor was transfected into HC cells treatedwith or without IL‐1β, which promoted a catabolic environment. After miR‐4498 inhibition, catabolism was downregulated, while anabolism was upregulated to nearly normal levels. The results were confirmed by RT‐qPCR, Western blotting (Figure 5F and G) and IF staining (Figure 5I, Figure S5C) in HC cells. Similar results were also identified in SW1353 cells (Figure S5A and B). The low expression of proteoglycan induced by IL‐1β stimulation was upregulated by treatment with the miR‐4498 inhibitor (Figure 5H). These results indicated that miR‐4498 inhibition mediated circSLC7A2 knockdown‐induced OA and partly attenuated the effects of by IL‐1β stimulation.

### 3.8 TIMP3 is a potential target of miR‐4498, and the overexpression of TIMP3 rescues the effects of miR‐4498 in OA

**FIGURE 6:**
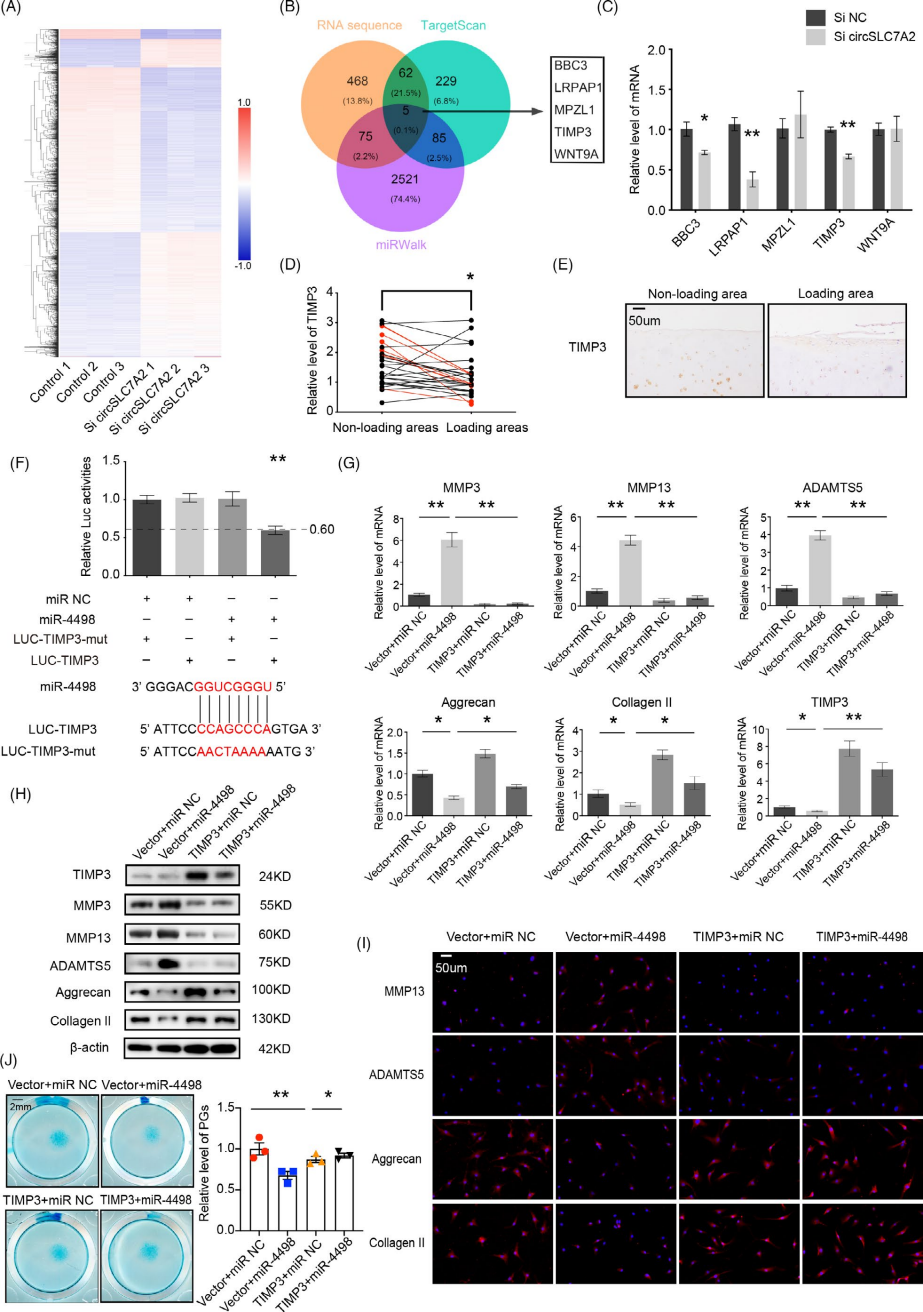
(A) The differential upregulation of mRNAs by circSLC7A2 knockdown was detected by RNA‐seq in HC cells compared with controls (n = 3). (B) Schematic flowchart showing the overlap of the downregulated mRNAs and target gene analysis of miR‐4498 from the TargetScan and miRWalk databases. (C) RT‐qPCR analysis of target genes in HC cells transfected with circSLC7A2 siRNA. (D & E) TIMP3 expression was downregulated in human medial cartilage compared with lateral cartilage from knee joints, as determined by RT‐qPCR (D), TIMP3 expressions in loading areas/non‐loading areas >2 were shown in red (*P < .05, comparative t test), and IHC (E), scale bar = 50 μm. (F) HEK‐293T cells were co‐transfected with miR‐4498 mimics or negative control and luciferase reporter constructs containing the TIMP3 WT or MUT 3′‐UTR. (G‐I) TIMP3, MMP3, MMP13, ADAMTS5, aggrecan and collagen II protein and mRNA levels in HC cells transfected with miR‐4498 mimics and sh‐TIMP3 lentivirus. The overexpression of TIMP3 can rescue miR‐4498 mimics, as evaluated by WB, RT‐qPCR, and IF. Nuclei were stained with DAPI (scale bar = 50μm). (J) Representative images of HC cells stained with Alcian blue that were transfected with miR‐4498 mimics and sh‐TIMP3 lentivirus, scale bar = 2 mm. Data are representative images of similar results obtained from three different donors (E, H, I and J) or presented as the mean ± SEM from three independent experiments (C, F and G). (*P < .05, **P < .01 vs control or as indicated by the Student's t test)

According to the competing endogenous RNA (ceRNA) hypothesis, miR‐4498 directly binds to target mRNAs, which may have a positive correlation with RNA‐seq data. Total RNA was extracted from chondrocytes (n = 3 donors per group) transfected with si‐circSLC7A2 or si‐NC to construct RNA‐seq libraries, and 325 potential genes met the significance threshold (fold change (SI/NC) > 1.5), calculated by DESeq2 (Figure 6A, Figure S6A). By comparing these genes using TargetScan and miRWalk, we identified five potential downstream targets (Figure 6B). To confirm the target of miR‐4498, we knocked down circSLC7A2 in HC cells and found that three target genes (BBC3, LRPAP1 and TIMP3) were downregulated (Figure 6C). To further investigate the function of these genes in chondrocytes, five siRNAs were specifically designed and transfected into HC cells. RT‐qPCR and Western blot analysis showed that TIMP3 had the most significant impact on MMP3, MMP13, ADAMTS5, aggrecan and collagen II expression (Figure S6B and C). TIMP3 expression in cartilage was confirmed by RT‐qPCR (Figure 6D) and IHC (Figure 6E), which indicated that TIMP3 was expressed at low levels in the loading areas. Moreover, stimulation by IL‐1β or TNF‐α in HC cells decreased TIMP3 and collagen II expression, which was accompanied by an increase in MMP13 (Figure S7A). In general, we found that TIMP3 plays an important role in ECM metabolism.

To determine whether miR‐4498 can directly interact with TIMP3, we co‐transfected miR‐4498 mimics and luciferase reporters containing the TIMP3 wild‐type 3′‐UTR sequence or a TIMP3 mutant 3’‐UTR sequence into HEK‐293 cells. The luciferase intensity of the wild‐type TIMP3 reporter was suppressed by miR‐4498 (Figure 6F), indicating that miR‐4498 can bind to TIMP3. In addition, the transfection of miR‐4498 mimics into HC and SW1353 cells reduced TIMP3 protein levels (Figure S7B). In contrast, the overexpression of TIMP3 reversed the influence of miR‐4498 on MMP3, MMP13, ADAMTS5, aggrecan and collagen II expression in both HC and SW1353 cells (Figure 6G‐I; Figure S7C and D). In addition, Alcian blue staining showed that the decrease in proteoglycans caused by miR‐4498 was rescued by TIMP3 **(**Figure 6J, Figure S7E). These results revealed that TIMP3 is a downstream target of miR‐4498.

### 3.9 CircSLC7A2 alleviates OA progression in a mouse ACLT model

**FIGURE 7:**
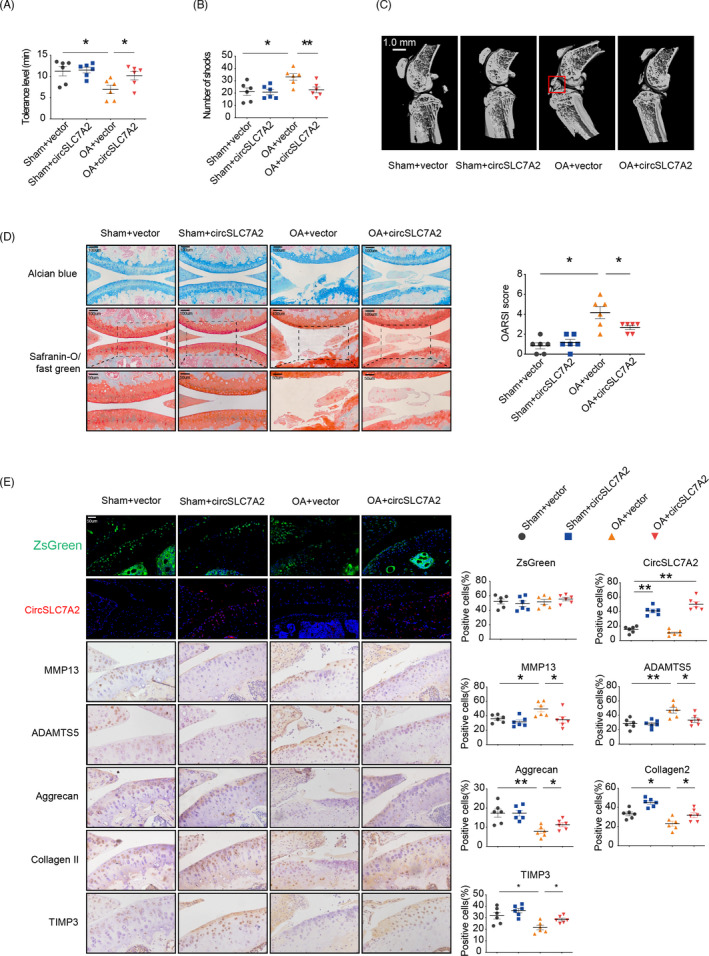
(A) Performance change of individual mice on a hotplate, described as the time of mice licking their hind limb or jumping was recorded, n = 6; *P < .05, **P < .01. (B) Performance change on treadmill of individual mice; numbers of mice given electric shocks were recorded, N = 6; *P < .05, **P < .01. (C) Micro‐CT images of OA mice. The red square indicated the formation of enormous osteophytes. (scale bar = 1 mm) (D) Alcian blue and Safranin O/fast green staining of ACLT‐induced OA mice injected with AAV negative control, WT AAV circSLC7A2 or MUT AAV circSLC7A2. Scale bar = 50‐100 μm. OARSI score was evaluated based on Safranin O/fast green staining, n = 6. *P < .05, **P < .01. (E) FISH and IHC staining of ACLT‐induced OA mice injected with AAV negative control, WT AAV circSLC7A2 or MUT AAV circSLC7A2. The expression of ZsGreen in mouse articular was detected. Representative images of IF are shown. Scale bar =50 μm, n = 6; *P < .05, **P < .01. Data are representative images of similar results obtained from 6 mice (C, D and E)

According to TargetScan, the miR‐4498/TIMP3 axis is highly conserved between mouse and homo sapiens. To investigate the function of circSLC7A2 in vivo, we successfully established a mouse model of knee OA by anterior cruciate ligament transection (ACLT). At 8 weeks post‐surgery, mice in the ACLT with circSLC7A2 injection group showed a significantly longer response times in the hotplate nociception analysis than those in the ACLT group (Figure 7A). Since the response time is a mark of algesia, this result indicated that ACLT induced joint pain, which was rescued by circSLC7A2. The treadmill experiment results demonstrated that the ACLT group was shocked more frequently than the ACLT with circSLC7A2 injection group, indicating that circSLC7A2 clearly rescued OA progression (Figure 7B). In addition, the formation of enormous osteophytes in the OA group was confirmed by the three‐dimensional reconstruction of micro‐CT images, while circSLC7A2 intra‐articular injection markedly suppressed osteophyte formation (Figure 7C). Safranin O/fast green staining and Alcian blue staining showed that cartilage surfaces in ACLT‐induced OA mice improved with the injection of circSLC7A2‐expressing virus (Figure 7D). The Osteoarthritis Research Society International (OARSI) score of the different groups was assessed using Safranin O/fast green staining. However, this effect was rescued by the introduction of the circSLC7A2‐expressing virus (Figure 7E). The function of circSLC7A2 in OA progression was confirmed by IHC (Figure 7E). Taken together, these results confirmed that circSLC7A2 plays a critical role in protection against degenerative changes in the cartilage matrix.

## 4 DISCUSSION

Although CircRNAs have been investigated for decades; however, their mechanistic roles in OA pathogenesis and progression remain largely unknown. CircRNAs are abundant in cells, plasma and even circulating exosomes and are more stable than their linear parental genes. Recent studies have reported that some circRNAs, which vary with external factors, can be used as biomarkers for disease diagnosis and treatment.^35^, ^36^ In the present study, a novel circRNA (circSLC7A2) that was highly expressed in healthy cartilage but significantly downregulated in OA clinical samples was identified (n = 28) (Figure 1). The results presented here elucidated the mechanism of circSLC7A2 regulation leading to changes in ECM properties as observed in OA and healthy cartilage tissues, indicating that circSLC7A2 may be a novel potential target for OA treatment.

CircSLC7A2 consists of a nucleotide sequence corresponding to exons 8‐10 of the SLC7A2 gene and is considered a key circRNA involved in OA. CircSLC7A2 is resistant to RNase R and is predominantly localized in the cytoplasm, where it functions to prevent OA progression. In this study, loss‐of‐function analysis revealed that circSLC7A2 downregulation induced cartilage destruction by increasing catabolic enzyme levels and decreasing anabolic enzymes (Figure 2). In addition, in chondrocytes and SW1353 cells, circSLC7A2‐knockdown induced apoptosis, which is involved in cartilage destruction and has been attributed to the initiation and progression of OA.^37^ We found that SLC7A2 was highly expressed in the representative loading areas (OA) compared with non‐loading areas (non‐OA) and played a crucial role in regulating the ECM by inhibiting collagen II and SOX9 and activating MMP13 and ADAMT5. Interestingly, we found a negative correlation between the expression levels and the exact opposite effects between linear SLC7A2 and circSLC7A2. As circRNAs can be regulated by RBPs^38^, ^39^ and both circRNAs and mRNAs are derived from pre‐mRNAs, we assumed that circSLC7A2 was post‐transcriptionally regulated by a specific RBP and the splicing of pre‐SLC7A2 may be a critical event in OA development. Previous studies have reported that several splicing factors, such as DHX9, ADAR1 and QKI, regulate circRNA biogenesis.^40^ Notably, FUS is predominantly a nuclear protein that is involved in multiple steps of RNA metabolism including transcription and mRNA transport for site‐specific translation and pre‐mRNA splicing.^41^ In the present study, we identified that FUS mediated the biogenesis of circSLC7A2 via interacting with ‘GUGGU’ motifs in the flanking intron regions of the circSLC7A2‐forming exons ^34^ and increasing the catabolic enzyme levels while decreasing the anabolic enzyme levels. Moreover, FUS was reported to be significantly downregulated in hypertrophic mouse chondrocytes cells, indicating that FUS may play a specific role in cartilage.^42^ Considering its role in circRNA splicing and ECM metabolism regulation, we deduced that FUS exerts its protective role by promoting the formation of circSLC7A2 but not linear SLC7A2 from pre‐SLC7A2 (Figure 3). Thus, we inferred that in the case of inflammatory stimulation or OA pathology, the binding of FUS to pre‐SLC7A2 was decreased, reducing the production of circSLC7A2, which further promoted OA progression. However, there could be other potential targets of FUS that function in cartilage, and these targets will need to be further investigated.

According to previous reports, circRNAs function primarily as ceRNAs, acting as an absorbing sponge for endogenous miRNAs, thereby upregulating the corresponding miRNA target genes.^43^ In addition, circRNAs are predicted to function as robust post‐transcriptional regulators of gene expression by interacting with many different RBPs and by acting as protein sponges.^17^, ^44^ The results of this study revealed that circSLC7A2 suppressed OA progression by binding to miR‐4498 and then promoting its degradation (Figure 4). Through gain‐ and loss‐of‐function analyses, we further discovered that miR‐4498 has an opposite effect to that of circSLC7A2 on OA progression (Figure 4). In addition, the inhibition of miR‐4498 rescued the inflammatory phenotype and apoptosis of chondrocytes and SW1353 cells caused by the loss of circSLC7A2 or by IL‐1β treatment (Figure 5). miRNAs have gained considerable attention as regulators of the ECM. For example, miRNA‐204 and mir‐211 have been reported to target distinct genes related to the development and progression of OA.^45^ miR‐98 contributes to ECM degradation and plays a role in regulating the vitality and functions of nucleus pulposus cells.^46^ Our results showed that compared with other miRNAs, miR‐4498 influenced an increased number of catabolic and anabolic genes, such as ADAMTS5 and aggrecan, and accelerated chondrocyte apoptosis, which has not been previously reported. As a major metalloprotease, ADAMTS5 was reported to be the most active protease because of its ability to degrade aggrecan,^47^ which is a large and highly complex macromolecule that is uniquely structured to fill space in the ECM of cartilage.^48^ Thus, in the present study, miR‐4498 showed a significant ability to regulate ECM metabolism and is likely to be an ideal target for OA treatment.

Our data further demonstrated that miR‐4498 functions through TIMP3 in the regulation of ECM metabolism. TIMP3 is a secreted 24‐kD protein that is a member of the tissue inhibitor of metalloproteinases family, which includes TIMP1, 2, 3 and 4.^49^ TIMPs play important roles in many tissues and are specific inhibitors of matrixins, which participate in controlling the activities of MMPs.^50^ TIMP1 is commonly found in cancer cells and is associated with a poor prognosis; TIMP2 inhibits the mitogenic response of human microvascular endothelial cells to growth factors; and TIMP4 is an MMP inhibitor in human platelets.^51^, ^52^, ^53^ Unlike the other members of the TIMP family, TIMP3 is well known for its unique ability to bind the ECM.^54^ TIMP3 effectively blocks aggrecan breakdown in IL‐1α‐stimulated cartilage in culture and plays an important role in regulating the ECM by broadly inhibiting MMPs, ADAMs and ADAMTSs.^55^, ^56^, ^57^ In this case, the circSLC7A2/miR‐4498 axis exhibited a wider spectrum of catabolic enzyme inhibition via TIMP3 regulation to prevent the loss of cartilage ECM during OA progression (Figure 6).

The intra‐articular injection of hyaluronic acid or corticosteroids is an effective method that has been widely used for clinical OA therapy in humans.^22^ CircRNAs are abundant, diverse and conservative across species, rendering them as potential biomarker candidates. During previous study, some researchers identified the corresponding conservative sequences of circRNA between human and mouse and then used animal sequences to complete animal experiments instead of human.^58^ However, a large number of circRNA do not exist conservative sequences in other species. In our study, the principium of in vivo experiments was to rely on the injected overexpressed human circSLC7A2 virus to bind to miR‐4498 in mice, which in turn affected the expression of TIMP3 in mice. Regarding miR‐4498, the binding sites of circSLC7A2 and miR‐4498 are conserved in human and mouse, both UGGGCUGG. Furthermore, TargetScan database showed that the combination of TIMP3 and miR‐4498 is conserved in human and rabbit. Thus, circSLC7A2‐miR‐4498/TIMP3 axis both works in human and mouse. Similar researches and animal models have been reported in previous study.^26^ Our animal model experiments showed that the intra‐articular treatment of ACLT mice with circSLC7A2 significantly alleviated OA progression, evidenced by the return of pain‐related behaviours to normal levels, reduced osteophyte formation and improved OARSI scores (Figure 7). Pain‐related behavioural assays are highly recommended in the study of OA animal models and are considered more sensitive and specific than osteophyte formation assays and OARSI scores.^59^ Because the ACLT model was induced in a single mouse joint, the measured behavioural responses were exclusively sensitive and specific for the injured joint and could be further quantitated for comparisons between groups.^60^ In addition, our OA animal model displayed pain behavioural characteristics highly similar to those observed in human patients with chronic OA, indicating its potential for direct translation.^61^

## CONFLICT OF INTEREST

The authors have declared that no competing interest exists.

## AUTHOR CONTRIBUTIONS

Ziang Hu, Shuying Shen and Shunwu Fan designed the experiments. Weiyu Ni, Chao Jiang Yizheng Wu and Haitao Zhang performed the experiments and acquired the data. Weiyu Ni, Lili Wang, Jasper HN Yik and Dominik R. Haudenschild analysed the data. Shunwu Fan, Jasper HN Yik and Ziang Hu supervised the project and wrote the manuscript.

## Supporting information

Supplementary Material

Supplementary Material

## Data Availability

The data that support the findings of this study are available from the corresponding author upon reasonable request.
