# Supplementary material for: CircSLC7A2 protects against osteoarthritis through inhibition of the miR‐4498/TIMP3 axis
Source: Cell Prolif. 2021 May 7;54(6):e13047. doi: 10.1111/cpr.13047 (PMC8168424; doi:10.1111/cpr.13047)
Supplement: Supplementary file 1 — Supplementary Material [file CPR-54-e13047-s002.docx]

**
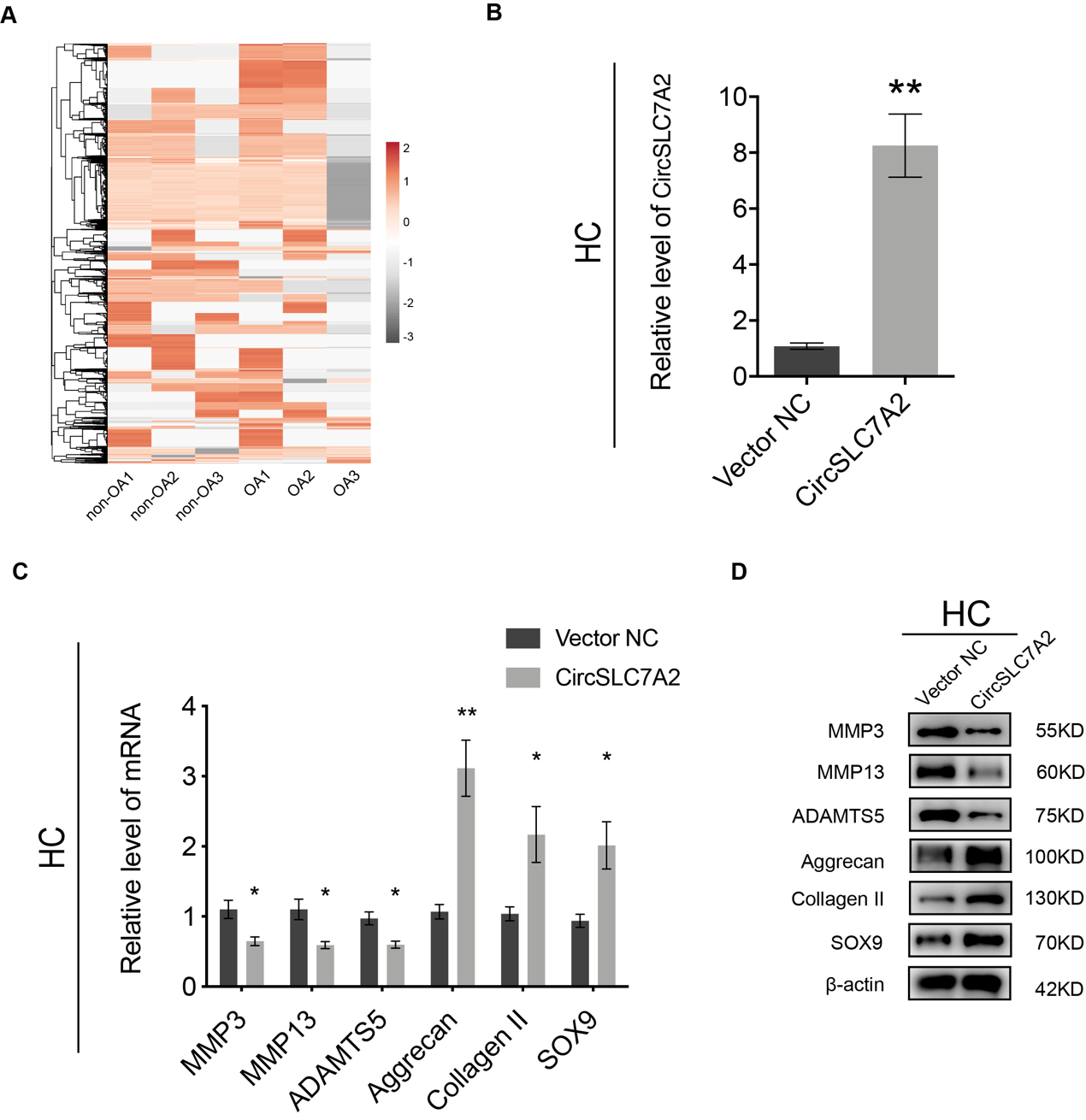
**

Figure S1. (A) CircRNA microarray based on OA and non-OA cartilage samples. (B) RT-qPCR quantification of circSLC7A2 levels in HC cells after circSLC7A2 overexpression. (C & D) RT-qPCR and western blot analyses of ECM associated proteins after circSLC7A2 overexpression. Data are from three independent experiments (mean ± SEM) (B and C) or representative images of three independent experiments with similar results (D) (*P < 0.05, **P < 0.01 vs control or as indicated by the Student’s t-test).


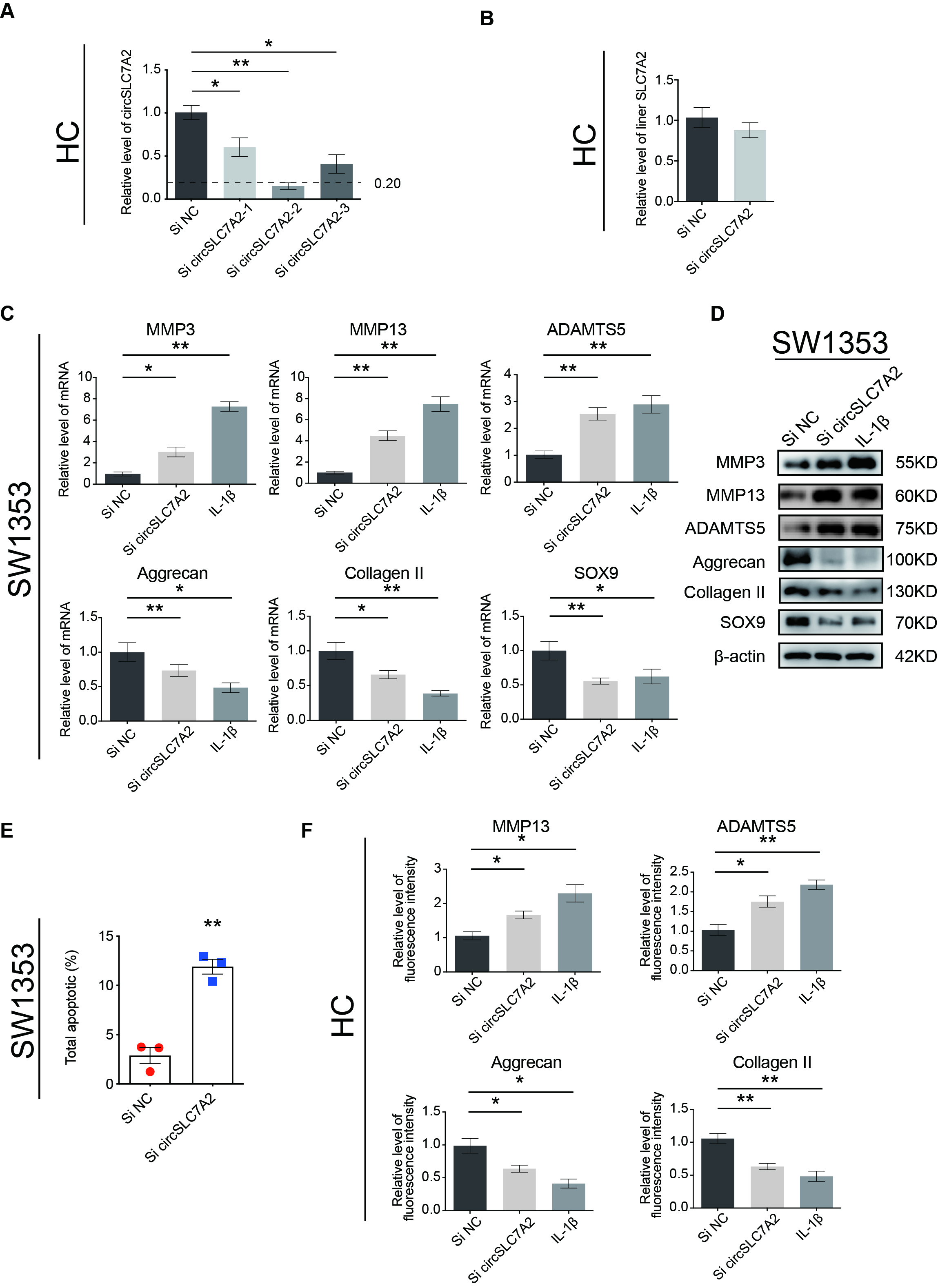


Figure S2. RT-qPCR analysis of (A) circSLC7A2 and (B) liner SLC7A2 mRNA expression in HC cells transfected with three different siRNAs or a scramble siRNA as negative control (NC). Gene expression levels in NC was arbitrarily set to 1. (C & D) MMP3, MMP13, ADAMTS5, Aggrecan, Collagen2 and SOX9 RNA levels in in SW1353 cells transfected with circSLC7A2 or treated with IL-1β; and western blotting. (E) Apoptosis flow cytometry detection were shown after Annexin V-FITC/propidium iodide (PI) dual staining. The transfection of circSLC7A2 and negative control in SW1353 cells. (F) Fluorescence intensity of IF of MMP13, ADAMTS5, aggrecan and Collagen2 in HC cells transfected with circSLC7A2 or treated with IL-1β. Data are from three independent experiments (mean ± SEM) (A, B, C, E and F) or representative images of three independent experiments with similar results (D) (*P < 0.05, **P < 0.01 vs control or as indicated by the Student’s t-test).

**
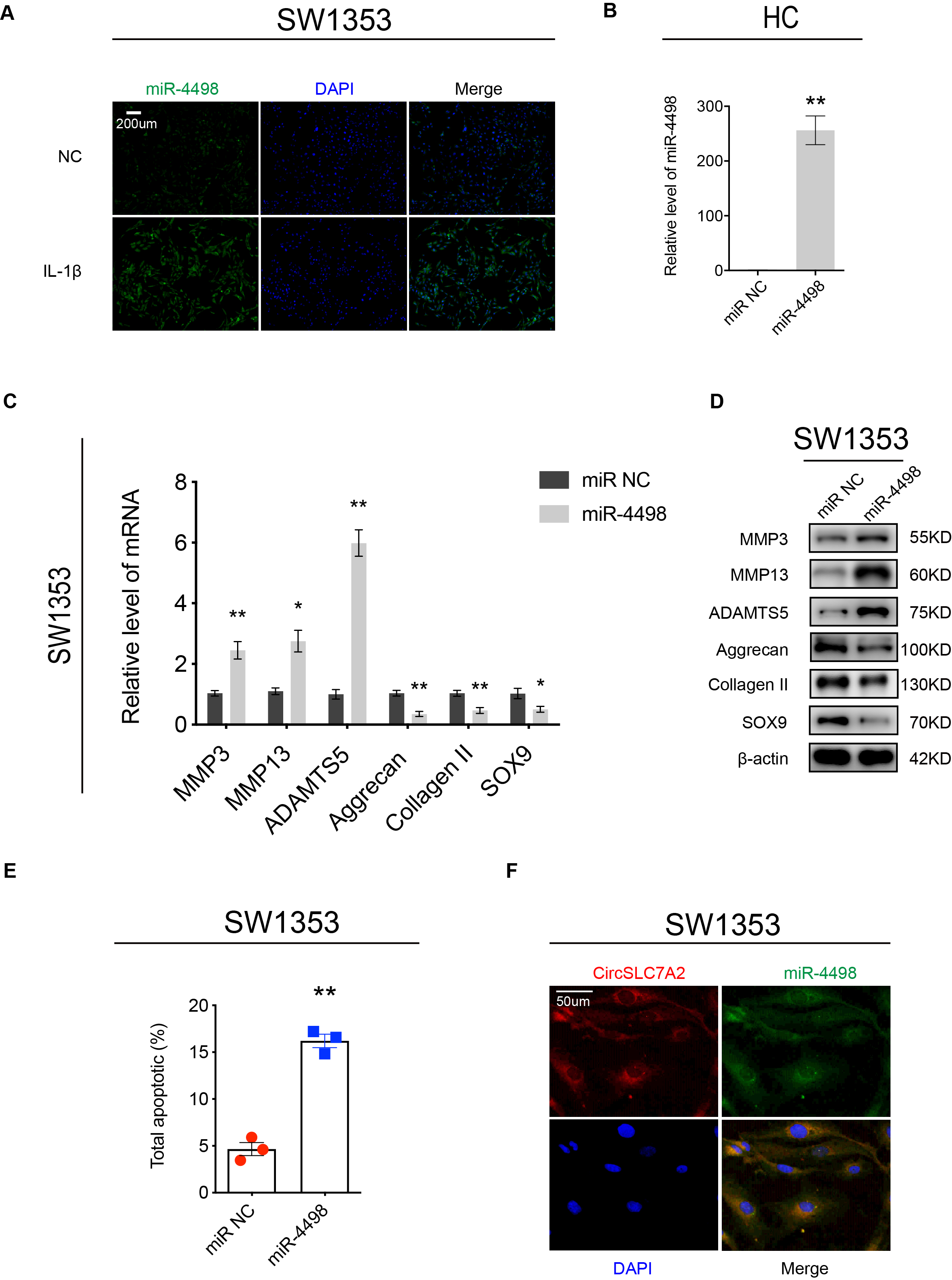
**

Figure S3. (A) miR-4498 was detected in SW1353 cells treated with IL-1β by FISH and its specific probe was labeled with Alexa Fluor 555; nuclei were stained with DAPI (scale bars, 200μm). (B) The transfection efficiency of miR-4498 mimics in HC cells was detected by RT-qPCR. (C & D) MMP3, MMP13, ADAMTS5 and aggrecan, Collagen2, SOX9 protein levels in SW1353 cells transfected with miR- negative control, miR-4498 as well as RT-qPCR results. (E) Apoptosis flow cytometry detection was shown after Annexin V-FITC/propidium iodide (PI) dual staining. The transfection of miR-4498 and negative control in SW1353. (F) miR-4498 was detected in SW1353 cells by FISH and its specific probe was labeled with Alexa Fluor 488; circSLC7A2 probes were labeled with Alexa Fluor 555; nuclei were stained with DAPI (scale bars, 50μm). Data are from three independent experiments (mean ± SEM) (B, C and E) or are representative images of three independent experiments with similar results (A, D and F) (*P < 0.05, **P < 0.01 vs control or as indicated by the Student’s t-test).

**
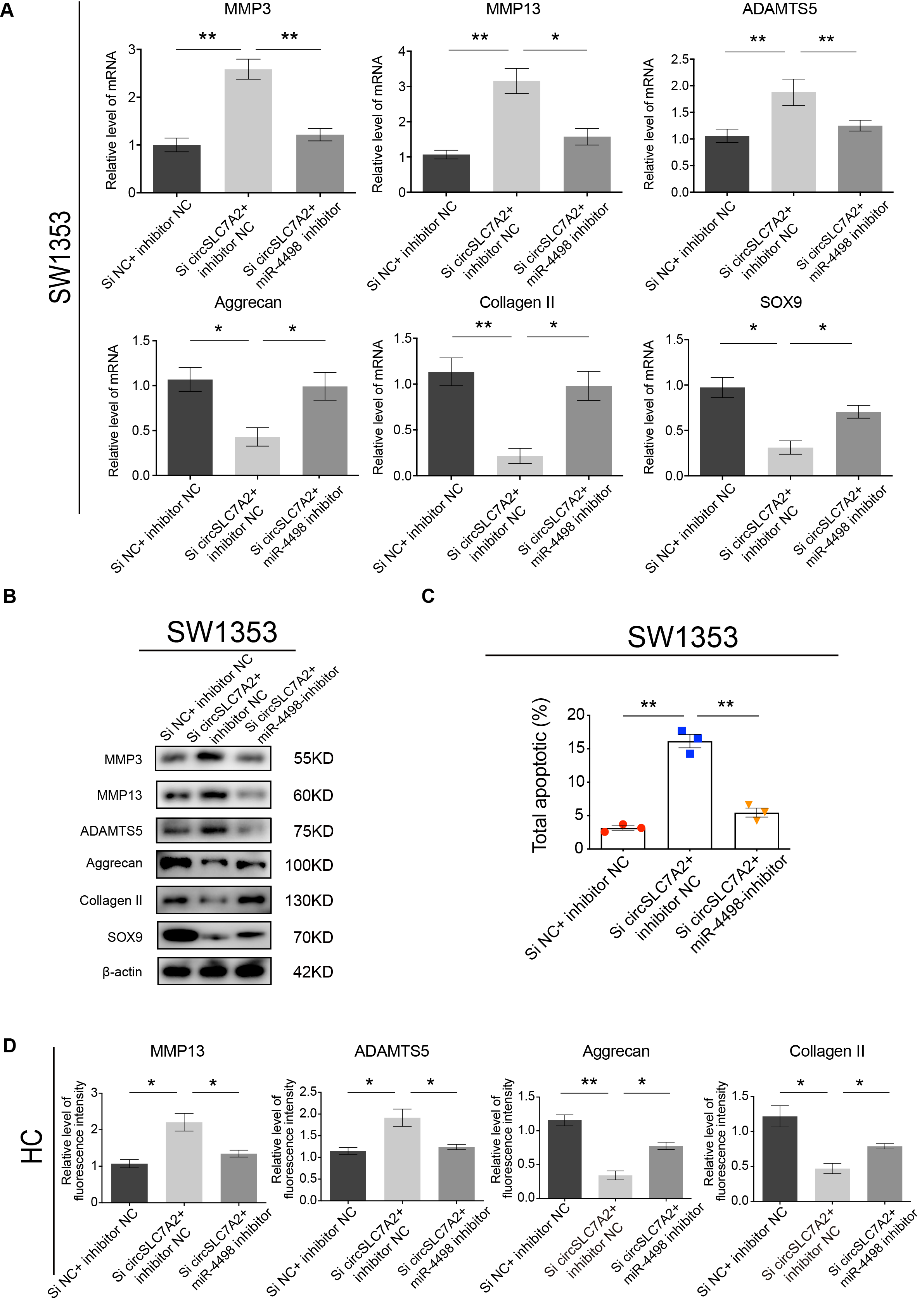
**

Figure S4. (A & B) MMP3, MMP13, ADAMTS5, Aggrecan, Collagen2 and SOX9 mRNA expression and protein synthesis in SW1353 cells that were co-transfected with si-circSLC7A2 and miR-4498-inhibitor. (C) Apoptosis flow cytometry detection was shown after Annexin V-FITC/propidium iodide (PI) dual staining. The co-transfection of si-circSLC7A2 with miR-4498 or negative control in SW1353. **P<0.01. (D) Fluorescence intensity of IF of MMP13, ADAMTS5, aggrecan and Collagen2 in HC cells co-transfected with si-circSLC7A2 and miR-4498-inhibitor. Data were from three independent experiments (mean ± SEM) (A, C and D) or were representative images of three independent experiments with similar results (B) (*P < 0.05, **P < 0.01 vs control or as indicated by the Student’s t-test).


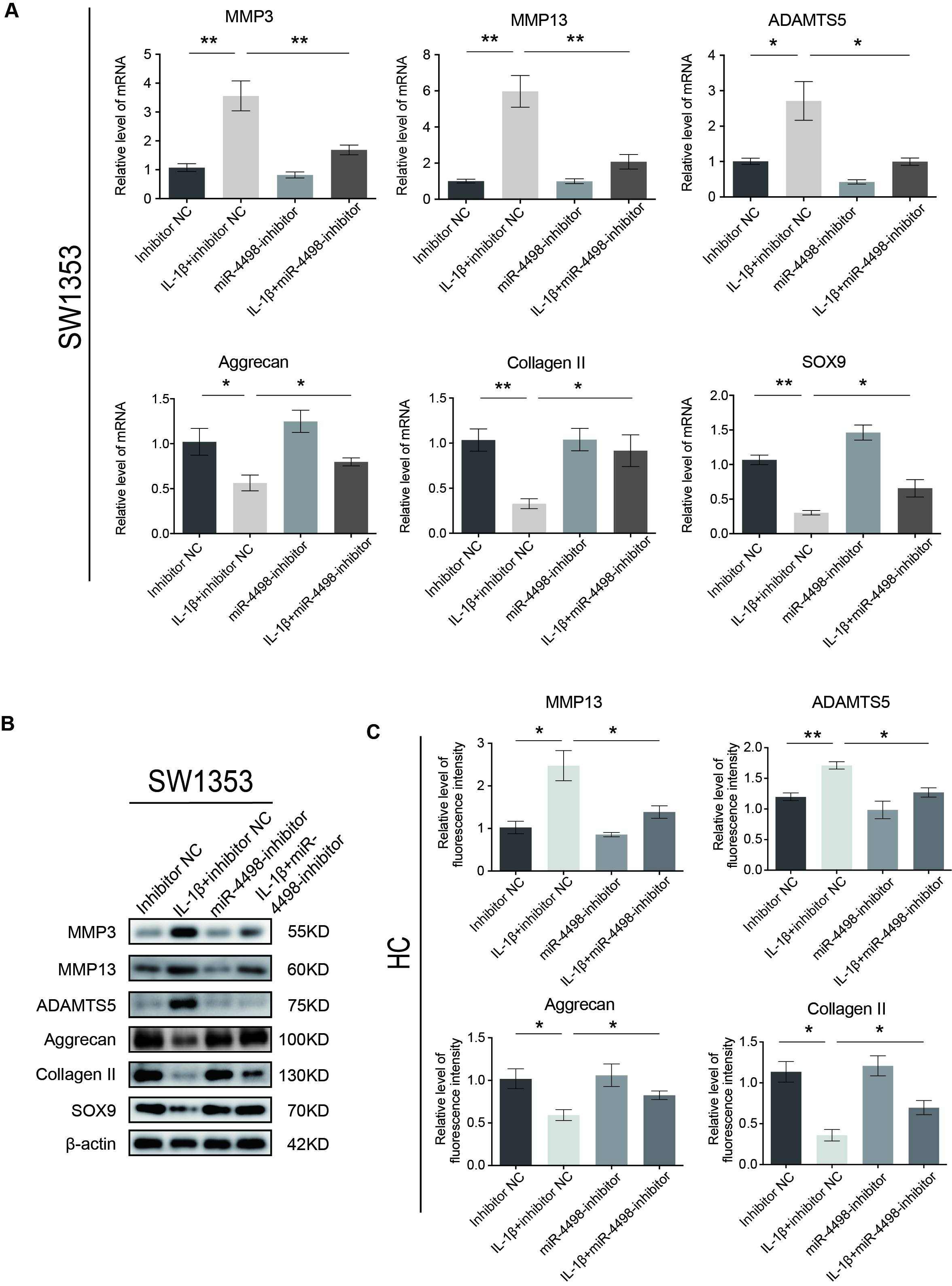


Figure S5. (A & B) SW1353 cells were transfected with miR-4498-inhibitor or negative control, and then exposed to IL-1β. qRT-PCR and WB for MMP3, MMP13, ADAMTS5, aggrecan, Collenge2 and SOX9 detection. (C) Fluorescence intensity of IF of MMP13, ADAMTS5, aggrecan and Collagen2 in HC cells transfected with miR-4498-inhibitor or negative control, and then exposed to IL-1β. Data were from three independent experiments (mean ± SEM) (A, C) or representative images of three independent experiments with similar results (B) (*P < 0.05, **P < 0.01 vs control or as indicated by the Student’s t-test).


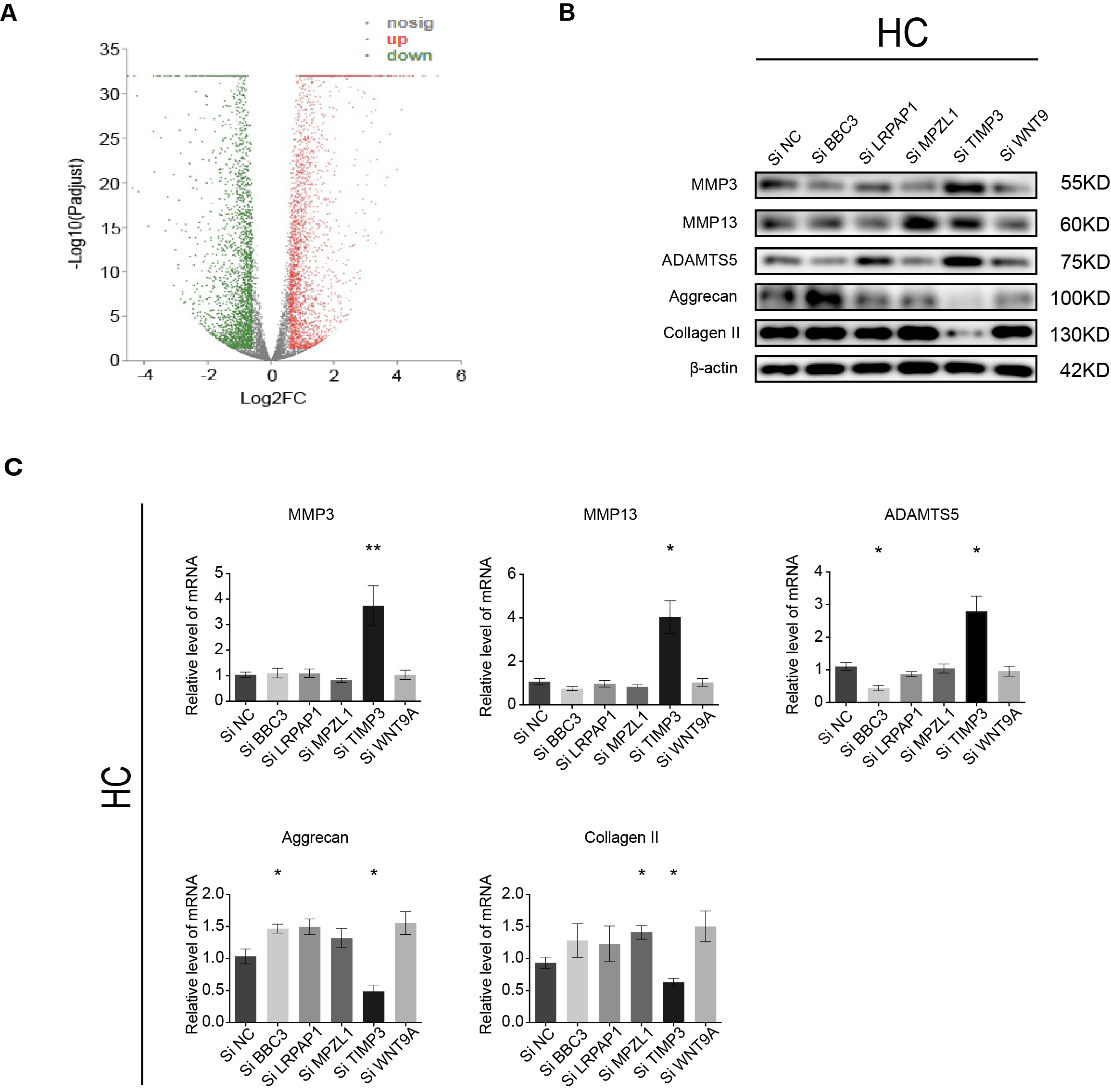


Figure S6. (A) Volcano plot of differentially expressed mRNAs following circSLC7A2 knockdown; genes were detected by RNA-seq in HC cells compared with controls. (B & C) qRT-PCR analysis of MMP3, MMP13, ADAMTS5, aggrecan and Collagen2 in SW1353 transfected with si-NC, si-BBC3, si-LRPAP1, si-MPZL1, si-TIMP3, si-WNT9; and Western Blotting. Data were representative images among similar results obtained from three different donors (B) or presented as mean ± SEM from three independent experiments (C) (*P < 0.05, **P < 0.01 vs control or as indicated by the Student’s t-test).


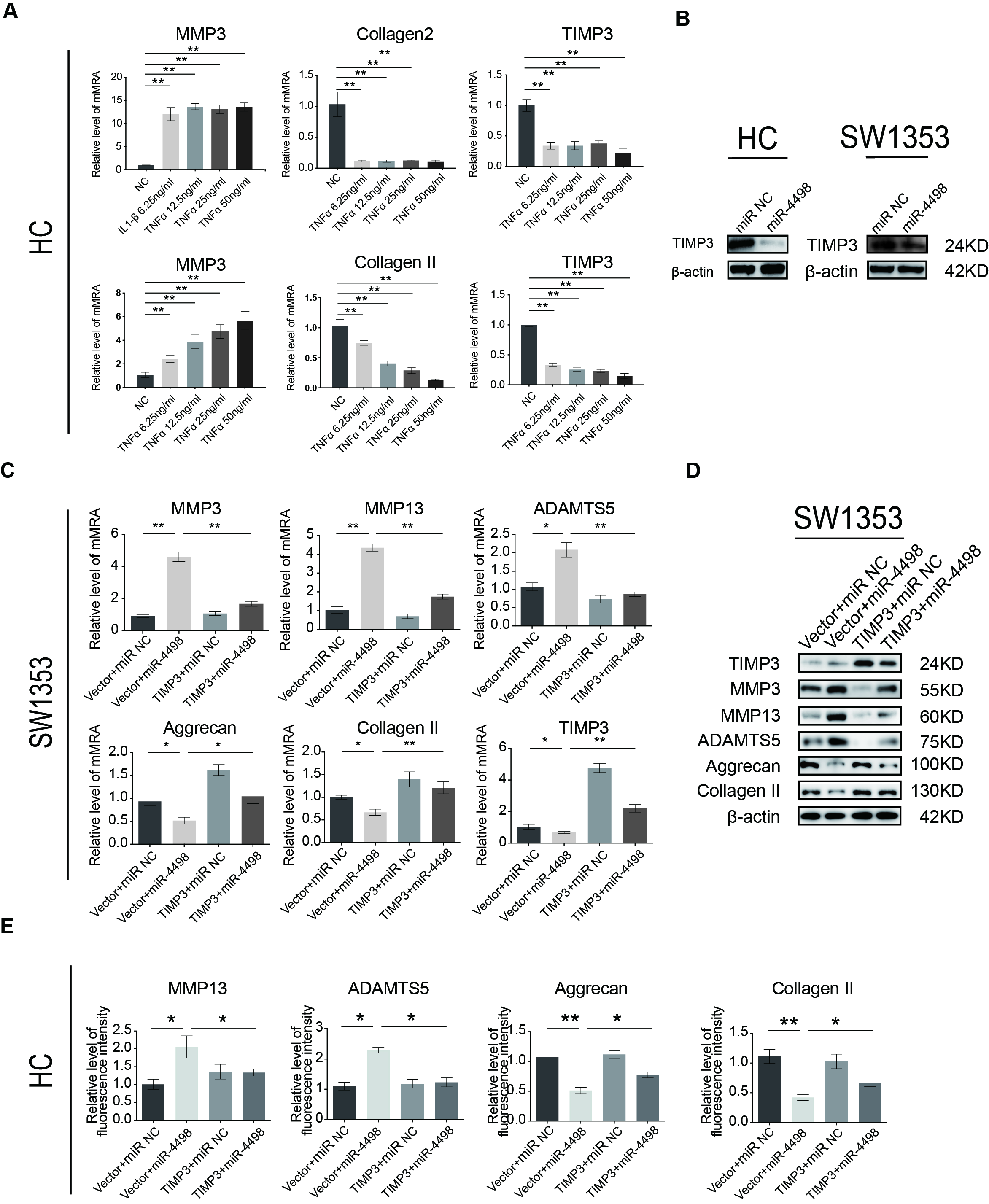


Figure S7. (A) Relative mRNA level of MMP3, Collagen2 and TIMP3 in HC cells treated with IL-1β and TNF-α in different concentration. (B) WB analysis of TIMP3 in HC cells and SW1353 transfected with miR-4498. (C & D) The mRNA and protein expression of TIMP3, MMP3, MMP13, ADAMTS5, aggrecan and Collagen2 in SW1353 transfected with miR-4498 mimics and sh-TIMP3 adenovirus. The overexpression of TIMP3 rescued on miR-4498 mimics were evaluated by qRT-PCR and WB. (E) Fluorescence intensity of IF of MMP13, ADAMTS5, aggrecan and Collagen2 in HC cells transfected with miR-4498 mimics and sh-TIMP3 adenovirus. Data were from three independent experiments (mean ± SEM) (A, C and E) or representative images of three independent experiments with similar results (B and D) (*P < 0.05, **P < 0.01 vs control or as indicated by the Student’s t-test).


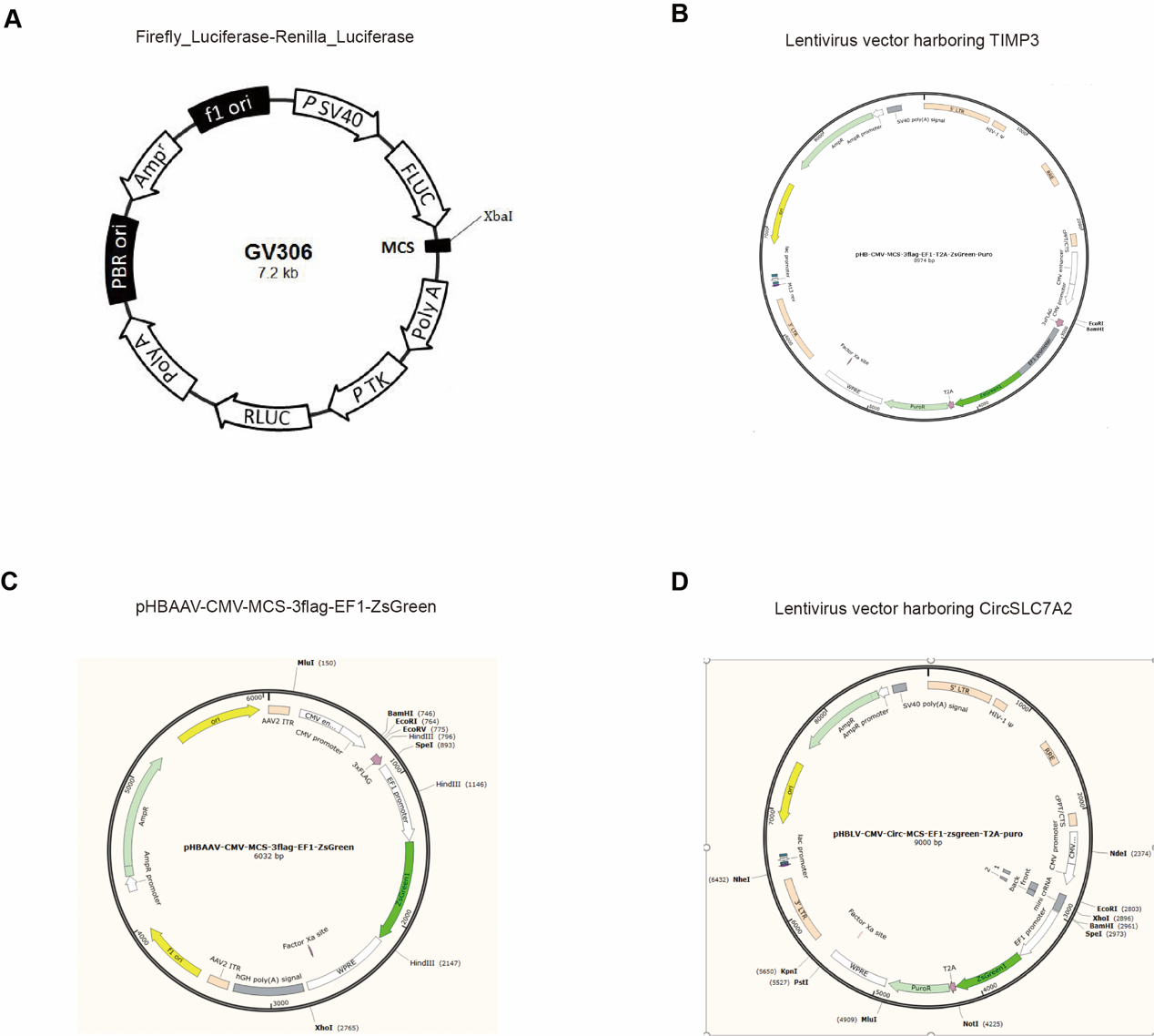


Figure S8. (A) The construct of reporter plasmids. (B) The construct of TIMP3 adenoviral vector. (C) The construct of AAV. (D) The construct of circSLC7A2 adenoviral vector.

Table S1: OA score from modified Pritzker OARSI score^61^

| Grade (key feature) | Associated criteria (tissue reaction) |
| --- | --- |
| Grade 0: surface intact, cartilage | Matrix: normal architecture  Cells: intact, appropriate orientation |
| Grade 1: surface intact | Matrix: superficial zone intact, oedema and/or superficial fibrillation (abrasion), focal superficial matrix condensation Cells: death, proliferation (clusters), hypertrophy, superficial zone  Reaction must be more than superficial fibrillation only |
| Grade 2: surface discontinuity | As above  +Matrix discontinuity at superficial zone (deep fibrillation)  ±Cationic stain matrix depletion (Safranin O or Toluidine Blue) upper 1/3 of cartilage  ±Focal perichondronal increased stain (mid zone)  ±Disorientation of chondron columns Cells: death, proliferation (clusters), hypertrophy |
| Grade 3: vertical fissures (clefts) | As above  Matrix vertical fissures into mid zone, branched fissures  ±Cationic stain depletion (Safranin O or Toluidine Blue) into lower 2/3 of cartilage (deep zone)  ±New collagen formation (polarized light microscopy, Picro Sirius Red stain) Cells: death, regeneration (clusters), hypertrophy, cartilage domains adjacent to fissures |
| Grade 4: erosion | Cartilage matrix loss: delamination of superficial layer, mid layer cyst formation Excavation: matrix loss superficial layer and mid zone |
| Grade 5: denudation | Surface: sclerotic bone or reparative tissue including fibrocartilage within denuded surface. Microfracture with repair limited to bone surface |
| Grade 6: deformation | Bone remodelling (more than osteophyte formation only). Includes: microfracture with fibrocartilaginous and osseous repair extending above the previous surface |

| Table S2: Primers and sequences used in this study | | |
| --- | --- | --- |
| Primers for qPCR | | |
| Gene | Forward primer: | Reverse primer |
| ADAMTS5 | GGGCACTGGCTACTATGTGG | CGTCACAGCCAGTTCTCACA |
| Aggrecan | CTACCAGTGGATCGGCCTGAA | CGTGCCAGATCATCACCACA |
| BBC3 | GCCAGATTTGTGAGACAA | CAGGCACCTAATTGGGCTC |
| hsa_circ_0005805 | CCTCGCGCTGTTTCTTGTTC | GCCATCAAAGCATGAAGGCTA |
| hsa_circ_0008590 | CCTCATATCGGGACCAGCAG | CCCAGGTTGTTAAAATCTTGTCAT |
| hsa_circ_0008365 | GTGCTCACCAGACTGGT | TTCACAATCTGATTGAAAAC |
| hsa_circ_0008667 | CCCATTTCTAATACTGAAGGTGTCC | TGCATTCCAACCAATCCCCA |
| hsa_circ_0002782 | GGTATCTCTACAGTGTTTAACTGCT | ATCCGAGGAGAGATGCCAGA |
| hsa_circ_0072568 | CCCTTGGATCCCATGACCAG | CAGACTGGCCAAGACCTGAG |
| hsa_circ_0030441 | AGCTTAAACCTGGCGTCATT | CTCTGTTACTGCCAATCCTGC |
| hsa_circ_0008012 | GTGGACTACCTGGTGCCTCT | CTGCCATTCTGAAGTTTGACCTG |
| hsa_circ_0006867 | TTACCTTGCCCACCAACTTCA | CACAGCAACTCTGTTTGCCAT |
| hsa_circ_0004662 | GGGAGCACGCTTACTACCTTC | GGGCTGTAACATCTCTCAGCAT |
| hsa_circ_0020093 | AATTGCGGCAGTCCAGATCA | GGTTCTGTTAACCTTGCCAACT |
| hsa_circ_0110251 | TCCAGGTGTACCAGCAGAAAC | TGAATGTCCCCAGGCTCAAC |
| hsa_circ_0001103 | CAGTGTGAGGTCCGGAATGT | GGACGACCCCTGGTTTCATT |
| hsa_circ_0001722 | TGGGAAGTTGGTAGCTCTGAAG | TTGGGACTTGTTGGCTTCCC |
| Collagen2 | ATGACAATCTGGCTCCCAAC | GAACCTGCTATTGCCCTC |
| FUS | ATGGCCTCAAACGATTATACCCA | GTAACTCTGCTGTCCGTAGGG |
| GAPDH | GGAGCGAGATCCCTCCAAAAT | GGCTGTTGTCATACTTCTCATGG |
| LRPAP1 | CGGAGGGTCAGGTCGTTTC | CAACTTCTCCATGCGGAACTC |
| MMP3 | CCTACAAGGAGGCAGGCAAG | CCCGTCACCTCCAATCCAAG |
| MMP13 | TCGGCCACTCCTTAGGTCTT | AAGTGGCTTTTGCCGGTGTA |
| MPZL1 | ACGCCAAAAGAAATCTTCGTGG | TCAACCCGCCAGTCGTACTA |
| miR-4498 | TGGGCTGGCAGGGCAAGT | UNIVERSAL REVERSE PRIMER (CWBIO) |
| miR-4741 | CGGGCTGTCCGGAGGGG | UNIVERSAL REVERSE PRIMER (CWBIO) |
| miR-6829-5P | TGGGCTGCTGAGAAGGGGC | UNIVERSAL REVERSE PRIMER (CWBIO) |
| miR-6852-5P | GCCCTGGGGTTCTGAGGACATG | UNIVERSAL REVERSE PRIMER (CWBIO) |
| miR-762 | GGGGCTGGGGCCGGGGC | UNIVERSAL REVERSE PRIMER (CWBIO) |
| SLC7A2 | GACCTTTGCCCGATGTCTGAT | AGCAGCGGCATAATTTGGTGT |
| Pre-SLC7A2 | CCTTCTGCTCAGGTCGCCTT | AGCGGCATAATTTGGTGTCTTC |
| SOX9 | GCTCTGGAGACTTCTGAACGA | CCGTTCTTCACCGACTTCCT |
| WNT9 | GGCAAGCATCTGAAGCACAAG | GCAGAAGCTAGGCGAGTCA |
| TIMP3 | CATGTGCAGTACATCCATACGG | CATCATAGACGCGACCTGTCA |
| U6 | CTCGCTTCGGCAGCACA | AACGCTTCACGAATTTGCGT |
| β-actin | AGAGCTACGAGCTGCCTGAC | AGCACTGTGTTGGCGTACAG |
| SiRNAs and miRNAs | | |
| hsa_circ_0005805_1 si | AAGTAGCCTTCATGCTTTG | |
| hsa_circ_0005805_2 si | AGTAGCCTTCATGCTTTGA | |
| hsa_circ_0005805_3 si | GCCTTCATGCTTTGATGGC | |
| hsa-miR-4498 | UGGGCUGGCAGGGCAAGUGCUG | |
| hsa-miR-4741 | CGGGCUGUCCGGAGGGGUCGGCU | |
| hsa-miR-6829-5P | UGGGCUGCUGAGAAGGGGCA | |
| hsa-miR-6852-5P | CCCUGGGGUUCUGAGGACAUG | |
| hsa-miR-762 | GGGGCUGGGGCCGGGGCCGAGC | |
| SiRNA NC | RiboBio | |
| Mimic NC | RiboBio | |
| Inhibitor NC | RiboBio | |
| hsa-miR-4498-inhibitor | CAGCACTTGCCCTGCCAGCCCA | |
| BBC3 si | GGGUCCUGUACAAUCUCAUTT | |
| FUS si | CAAGCAGATTGGTATTATT | |
| MPZL1 si | GGAAGTATATACGCCAAAA | |
| TIMP3 si | GCCTTAAGCTGGAGGTCAA | |
| LRPAP1 si | GCAAACUGGGCAAACGCUATT | |
| WNT9A si | GCAGCAAGTTCGTCAAGGA | |
| SLC7A2 si | GTGGCAAACTGGAAGATTA | |
| Probes for FISH | | |
| Cy3-circSLC7A2 | AAAGGCCATCAAAGCATGAAGGCTACTTT | |
| Alexa flour 488-miR-4498 | CAGCACTTGCCCTGCCAGCCCA | |
| Probes for RNA pulldown | | |
| hsa_circ_0005805 | CAAAGCATGAAGGCTACTTT | |
| Pre-SLC7A2 | TGATGTACCTTCCCAAAACA | |
| NC probe | Thermo | |
