# Supplementary material for: CircSLC7A2 protects against osteoarthritis through inhibition of the miR‐4498/TIMP3 axis
Source: Cell Prolif. 2021 May 7;54(6):e13047. doi: 10.1111/cpr.13047 (PMC8168424; doi:10.1111/cpr.13047)
Supplement: Supplementary file 2 — Supplementary Material [file CPR-54-e13047-s001.docx]

Experimental procedures

**Isolation and culture of human chondrocyte***s*

Cartilage was harvested from the discarded tissues of patients (males, aged 60-90 years) who underwent total knee replacement surgery. Consent was obtained from the patients prior to tissue collection, according to a protocol approved by the Ethics Committee of Sir Run Run Shaw Hospital (Zhejiang, China). For each specimen, relatively healthy cartilage (with no apparent lesions) from the lateral femoral condyle was harvested separately from the osteoarthritic cartilage (with cartilage lesions) at the medial femoral condyle. The harvested cartilage was either used for RNA extraction or processed further to isolate chondrocytes. To isolate chondrocytes, cartilage was cut into small pieces and treated with 0.25% pronase (Sigma, Louis, MO, USA) for 30 min followed by 0.2% collagenase type II (Invitrogen, Carlsbad, CA, USA) for 4 h at 37°C. The digest was filtered through a 0.075 mm cell strainer, and the cells were cultured overnight in Dulbecco’s modified Eagle’s medium (DMEM) containing 10% foetal bovine serum (FBS) (Thermo Fisher Scientific, Waltham, MA, USA) at 37°C in 5% CO_2_. Cellular debris was then removed by washing with sterile phosphate-buffered saline (PBS). Chondrocytes were continuously cultured and used for experiments within three passages.

**Cell lines and culture**

SW1353 and HEK-293 cells were obtained from the American Type Culture Collection (Manassas, VA, USA) and maintained in DMEM containing 10% FBS at 37°C in 5% CO_2_.

**Nucleic acid electrophoresis**

gDNA was purified from human chondrocytes seeded in 6-well plates (1×10^5^ cells per well) using an Ultrapure RNA kit (CWBIO, Beijing, China) according to the manufacturer’s instructions. gDNA and cDNA were amplified with PCR MasterMix (Yeason, Shanghai, China) with primers for SLC7A2, circSLC7A2, and β-actin. DNA molecular markers (Yeason, Shanghai, China) and gDNA or cDNA (generated from total RNA described below) PCR products (1 µg per well) were loaded onto 2% agarose gels in Tris-acetate-ethylenediaminetetraacetic acid (EDTA) (TAE) buffer and subjected to electrophoresis at 120 V for 30 min. The DNA bands were visualised under UV irradiation.

**FISH**

Cy3-labelled specific probes for detecting circSLC7A2 and 488-labelled locked nucleic acid miR-4498 probes were designed and synthesised by RiboBio (Guangzhou, China). Chondrocytes or SW1353 cells were grown on coverslips and treated with 10 ng/mL IL-1β (PeproTech, Beijing, China). After 48 h, the cells were fixed with 4% paraformaldehyde for 10 min and washed three times with PBS. Cells were permeabilized with 0.1% Triton X-100 in PBS for 5 min and then washed three times with PBS. FISH signals were detected using a FISH kit (RiboBio, Guangzhou, China), according to the manufacturer’s guidelines. Briefly, cells were incubated with circSLC7A2 or miR-4498 probes overnight at 37°C. The cells were then washed three times with washing buffer at 42°C. Nuclei were stained with 4, 6-diamidino-2-phenylindole (DAPI) (Life Technologies, Carlsbad, CA, USA) for 10 min at room temperature. Fluorescence images were acquired using a fluorescence microscope (BX51TRF; Olympus, Tokyo, Japan).

**SiRNAs, vector construction, and** **lentivirus overexpression**

SiRNA targeting circSLC7A2, BBC3, SYT7, LRPAP1, MAPKAPK3, and TIMP3 and miRNA mimics/inhibitors were designed and synthesised by RiboBio (Guangzhou, China). Synthetic miRNA mimics function by simulating miRNAs. miRNA inhibitors function by binding to the miRNA complementary strand. SiRNAs and miRNA mimics/inhibitors were transfected into cells seeded in 6- or 24-well plates with Lipofectamine iMax (Invitrogen, Carlsbad, CA, USA) (1 µl per 10^5^ cells). To construct the lentiviral vector construct harbouring TIMP3 (pDC315-TIMP3), the TIMP3 cDNA sequence was inserted into the pDC315-EGFP vector (Hanbio Co. Ltd, Shanghai, China) under the control of the mouse cytomegalovirus promoter. Two micrograms each of the pDC315-TIMP3 or pDC315-EGFP plasmids were co-transfected with packing plasmid (pCL10A1) into HEK-293 cells seeded in a 100 mm petri dish (10^7^ cells per dish) using Lipofectamine 3000 (Invitrogen, Carlsbad, CA, USA) according to the manufacturer’s protocols. After 48 h, the medium containing lentivirus expressing TIMP3 (Ad-TIMP3) or GFP (Ad-GFP) was purified with a 0.45-um strainer (Millipore, Billerica, Massachusetts, USA). After filtration, the medium was added to HC or SW1353 cells seeded in a 100 mm petri dish (5×10^6^ cells per dish). After infection, the cells were selected by treatment with 2 µg/mL puromycin (Gibco, Grand Island, NY, USA) for 1 week, and the surviving cells were continuously cultured before being plated for experiments. The construction of circSLC7A2 overexpression lentivirus used another lentivirus vector and the rest procedure was performed. All the sequences are shown in Supplementary Table S2. The lentivirus vector constructs are shown in Supplementary Fig S8.

**RNA extraction,** **RNase R treatment, and RT-qPCR**

Total RNA was extracted and purified from human cartilage or cultured chondrocytes using TRIzol reagent (Accurate Biotechnology, Hunan, China) reagent and an Ultrapure RNA kit (CWBIO, Beijing, China) according to the manufacturer’s instructions. For the enrichment of circRNAs, 2 µg of total RNA was incubated with or without 3 U/µg of RNase R (Epicentre Technologies, Madison, WI, USA) in RNase R reaction buffer for 20 min at 37°C. cDNA was synthesised from 1 µg of total RNA using a Hieff qPCR SYBR Green Master Mix (Yeason, Shanghai, China). RT-qPCR was performed with gene-specific primers (Supplementary Table S2) and SYBR® Green Premix Pro Taq HS qPCR Kit (Accurate Biotechnology, Hunan, China) using an ABI 7500 Sequencing Detection System (Applied Biosystems, Foster City, CA, USA) according to the manufacturer’s protocols. For the miRNA analyses, miRNAs were purified separately from cells using a miRNA Purification Kit (CWBIO, Beijing, China) and treated with DNase I to eliminate gDNA. cDNA was synthesised using an miRNA cDNA Synthesis Kit (CWBIO, Beijing, China), and RT-qPCR was performed as described above. The relative expression levels of the PCR products were calculated using the 2^-ΔΔCt^ method. The individual gene expression levels were normalised to those of β-actin for mRNA analysis or U6 for miRNA analyses.

**Luciferase reporter assays**

HEK-293T cells were seeded in 24-well plates (2×10^4^ cells per well) and cultured for 24 h. To evaluate the ability of circSLC7A2 to bind target miRNAs, cells were co-transfected with a mixture of reporter plasmids (pGL3-Firefly_Luciferase-Renilla_Luciferase containing the circSLC7A2 sequence) or mutant reporter plasmids, various miRNAs or control mimics (RiboBio, Guangzhou, China). The mutant circSLC7A2 sequence was designed according to the miRNA binding sites on circSLC7A2, which were predicted by the bioinformatics programs miRanda and TargetScan. When reporter plasmids bound to miRNAs, the activities of firefly luciferase would be inhibited but Renilla luciferase would not be influenced. To evaluate the ability of circSLC7A2 to bind miR-4498, the cells were assigned to the following experimental groups: (1) mutant reporter plasmids and miRNA mimic negative control; (2) mutant reporter plasmids and miRNA-4498 mimics; (3) luciferase-circSLC7A2 and miRNA-4498 mimic negative control; and (4) luciferase-circSLC7A2 and miRNA-4498 mimic. After 48 h, the firefly and Renilla luciferase activities were detected using the Luciferase Assay Reagent (Yeason, Shanghai, China) according to the manufacturer’s protocol. Firefly luciferase activity was normalised to Renilla luciferase activity to determine the ratio and the fold-change was calculated by comparing the ratio of the experimental group (groups 2, 3 and 4) to that of the negative control group (group 1). The ability of miR-4498 to bind target mRNAs was also evaluated according to the method described The reporter plasmid constructs are shown in Supplementary Fig S8.

**Western blotting**

Chondrocytes or SW1353 cells were seeded in 6-well plates (1×10^5^ cells per well) and transfected with siRNAs, miRNA mimics, or miRNA inhibitors. As a control, cells were treated with 10 ng/mL IL-1β (PeproTech, Beijing, China) for 48 h. At 48 h post-transfection, cells were lysed with RIPA lysis buffer (Fudebio, Hangzhou, China) and total protein was quantified using a bicinchoninic acid analysis kit (Fudebio, Hangzhou, China). Equal amounts of protein (20 µg per well) were loaded onto SDS-polyacrylamide gels (10%) for electrophoresis and the resolved bands were electroblotted onto polyvinylidene fluoride membranes. Membranes were blocked in 5% skimmed milk and then probed with antibodies specific for MMP3, MMP13, ADAMTS5, collagen II, Sox9 (all at 1:1000 Abcam), aggrecan (1:1000, ABclonal), β-actin (1:2000, Cell Signalling Technology), or TIMP3 (1:1000, ABclonal) overnight at 4 °C. Membranes were washed three times with TBS containing 0.1% Tween 20 and were then incubated with mouse or rabbit secondary antibody conjugated to horseradish peroxidase (HRP) (1:5000, Fudebio, Hangzhou, China) at room temperature for 1 hour. Protein bands were detected using FDbio-Femto ECL (Fudebio, Hangzhou, China) substrates and a chemiluminescence system (Bio-Rad, USA). The signal intensities of the protein bands were quantified using the Image Lab Software.

**IF microscopy**

Chondrocytes were grown on coverslips and treated with 10 ng/mL IL-1β (PeproTech, Beijing, China) or transfected with siRNAs, miRNA mimics or miRNA inhibitors. After 48 h, chondrocytes were fixed with 4% paraformaldehyde for 10 min and washed three times with PBS. Cells were permeabilized with 0.1% Triton X-100 for 5 min, washed three times with PBS, and then blocked in 10% goat serum for 1 hour. Cells were incubated with MMP13, ADAMTS5, aggrecan and collagen II antibodies (1:200; Abcam) overnight at 4 °C. After washing three times with PBS, the cells were incubated with goat anti-rabbit IgG conjugated to Cy5 (1:200; Fudebio, Hangzhou, China) in PBS for 1 h at room temperature. Nuclei were stained with DAPI (Yeason, Shanghai, China) for 10 min at room temperature. Fluorescence signals were detected using a fluorescence microscope (BX51TRF; Olympus, Tokyo, Japan).

**Flow cytometry**

Chondrocytes or SW1353 cells were seeded in 6-well plates (1×10^5^ cells per well) and transfected with siRNAs, miRNA mimics or miRNA inhibitors. After 48 h, apoptosis was determined using an Annexin V-FITC/PI Apoptosis Kit (BD Biosciences, Franklin Lakes, NJ, USA). Briefly, the cells were detached with 0.05% trypsin and washed twice with PBS. The cells were then incubated with annexin V-FITC and propidium iodide for 15 min prior to analysis with a flow cytometer (BD FACSCANTO II; BD Biosciences, San Jose, CA, USA) and FlowJo software to evaluate apoptosis.

**Alcian blue staining**

To assess chondrogenic differentiation glycosaminoghycans deposition, 1.5×10^5^ chondrocytes were resuspended in 10 μl of control medium and seeded as micromasses in the middle of a 24-well plate. Then, 0.5 mL DMEM containing 10% FBS was added to each well and incubated for 1 h at 37 °C. The medium was refreshed every other day. After 9 days micromasses were stained with Alcian blue. Chondrocytes were fixed with 4% paraformaldehyde for 20 min and stained with 1% Alcian blue (Sigma, St Louis, MO, USA) for 10 min. The relative levels of proteoglycans were determined by measuring the intensity of Alcian blue staining or toluidine blue staining using ImageJ (1.48, NIH, Bethesda, MD, USA).

**Pull-down assay**

A Pierce^TM^ Magnetic RNA-Protein Pull-Down Kit (Thermo, Waltham, MA, USA) was used for this experiment as indicated. Briefly, 2 × 10^7^ SW1353 cells were collected, lysed and sonicated. The beads were washed twice with 20 mM Tris before use. Labelled RNA (RiboBio, Guangzhou, China) or negative control was incubated with 50 μL of streptavidin magnetic beads in RNA capture buffer at room temperature for 30 min. The samples were incubated with labelled RNA at 4°C overnight. After washing three times with wash buffer, the samples were eluted with Biotin Elution Buffer after 15 min of incubation at 37°C. The RNA and protein complexes bound to the beads were analysed using RT-qPCR and western blotting. The probe sequences are listed in Supplementary Table S2.

**Silver staining**

Silver staining was performed using a Fast Silver Stain Kit (Beyotime, Haimen, China), as described in the protocol. Briefly, the proteins obtained from the RNA pulldown experiment (20 µg per well) were loaded onto SDS-polyacrylamide gels (12%) for electrophoresis. After being fixed with stationary liquid (50 mL ethanol, 10 mL acetic acid, and 40 mL double distilled H2O) for 1 h, the gels were washed with 30% ethanol for 10 min and washed twice with double distilled H2O. The gels were incubated with silver staining buffer for 10 min and washed twice with double-distilled H2O. Next, the gels were stained with a chromogenic agent until clear bands were visible, after which they were washed with the corresponding elimination agent.

**Mass spectrometry a****nalysis**

From the protein obtained from the RNA pulldown experiment, a sample of 120 µg was labelled with a TMT-10plex Isobaric Label Reagent Set plus TMT11-131C Label Reagent (A34808; Thermo Fisher Scientific). After drying and resuspension, the sample was loaded onto an Ultimate 3000 nanoflow liquid chromatography system (Thermo Scientific, USA) connected to a hybrid Q-Exactive HFX mass spectrometer (Thermo Scientific, USA). The results were analysed using a Q-Exactive coupled mass spectrometer (Thermo Fisher Scientific). All the steps were performed according to the manufacturer’s instructions.

**RIP assay**

Ago2 is a critical protein for miRNAs to sponge circRNAs and can form complexes with miRNAs and circRNAs. In total, 2 × 10^7^ SW1353 cells were transfected with miR-4498 or miR-NC. After 48 h, RIP was performed using a Magna RIP RNA-Binding Protein Immunoprecipitation Kit (Millipore, Billerica, MA, USA) according to the manufacturer’s protocol. Briefly, the cells were lysed in complete RIP lysis buffer. The resulting cell lysates were incubated with 5 μg of anti-Ago2 or control IgG antibody at 4°C overnight. Total RNA was then isolated for the detection of circSLC7A2 by RT-qPCR as described above. To evaluate the ability of pre-SLC7A2 to bind FUS, 2 × 107 SW1353 cells were collected, lysed, and incubated with 5 μg of anti-FUS or control IgG antibody at 4°C overnight. Total RNA was isolated for the detection of pre-SLC7A2 using RT-qPCR.

**RNA isolation and library construction** **for RNA-seq**

To identify the target genes of circSLC7A2, chondrocytes (n=3 donors per group) were seeded in 6-well plates (1 × 10^5^ cells per well) and transfected with siRNA against circSLC7A2 (si-circSLC7A2) or negative control (si-NC). Total RNA was purified with TRIzol reagent (Accurate Biotechnology, Hunan, China) and an Ultrapure RNA kit (CWBIO, Beijing, China). RNA-seq libraries were constructed using an Illumina TruSeq Small RNA Sample Prep Kit (Illumina Inc., San Diego, CA, USA) according to the manufacturer's instructions. RNA-seq was performed on an Illumina NovaSeq platform at Shanghai Majorbio Bio-Pharm Technology Co. Ltd. (Shanghai, China). A total of 325 unique genes were identified between the control and samples that met the significance threshold (E-value ≤ 10^−20^) and fold change threshold (SI/NC) >1.5, as calculated by DESeq2.

**Bioinformatics analysis**

The miRNA targets of circSLC7A2 were predicted using the following three bioinformatics programs: TargetScan, RNAhybrid, and miRanda. The filtering criteria were as follows: (i) P value ≤ 0.05, and (ii) number of estimated binding sites > 1. The mRNA targets of miR-4498 were predicted using the following two bioinformatics programs: TargetScan and miRWalk. The filtering restrictions were as follows: (i) site counts of 8mer ≥ 1, and (ii) longest consecutive pairings > 8.

**ACLT mouse model of OA**

Animal assays were approved by the Committee for Animal Care and Use of Sir Run Run Shaw Hospital (Zhejiang, China). C57BL/6J mice (12 weeks; n=8 mice per group) were purchased from the Shanghai SLAC Laboratory Animal Co. Ltd. (Shanghai, China). Mice were randomly assigned to the following experimental groups: (1) sham control with mock injection; (2) sham control with circSLC7A2 injection; (3) ACLT with mock injection; and (4) ACLT with circSLC7A2 injection. Animals were anaesthetized with 75 mg/kg ketamine and 10 mg/kg xylazine and subjected to unilateral ACLT procedures. Briefly, a skin incision was made to expose the patella, which was then dislocated laterally using tweezers under a microscope. The knee was placed in full flexion to expose the ACL, which was then transected with a needle. The incision was sutured using 3-0 silk sutures. The sham group received a skin incision and suturing without patellar dislocation or ligament transection.

**Generation and intra-articular injection of AAV expressing circSLC7A2**

The full-length circSLC7A2 DNA sequence was cloned into the adeno-associated virus serotype 2 (AAV2) vector pAV-FH (Hanbio Co. Ltd, Shanghai, China). The AAV2 vector pAV-C was used as a negative control. CircSLC7A2 or control plasmids were co-transfected with packaging plasmids (pHBAAV-CMV-MCS-3flag-EF1-ZsGreen) into HEK-293 cells using Lipofectamine 3000. After 72 h, the culture media were collected, and AAV was purified by PEG8000 precipitation. The viral titre was determined using RT-qPCR. At 1-week post-surgery, the ACLT and control mice (n=8 mice per group) were randomly assigned to receive intra-articular injections of either 10 µL of control virus or AAV expressing circSLC7A2 (1×10^13^ vg/ml). At 8 weeks post-surgery, mice were subjected to behavioural testing. Then, the mice were sacrificed, and their knees were processed for micro-CT analysis and IHC. The AAV construct is shown in Supplementary Fig S8.

**Behavioural test****ing**

At 8 weeks post-surgery, thermal hyperalgesia was assessed using the hotplate assay and joint pain was assessed by the treadmill experiment. The animals were acclimated in the procedure room for at least 30 min prior to the assessment. To assess thermal hyperalgesia, mice were individually placed on a hotplate (IITC, Los Angeles, CA, USA) at 55°C and the time elapsed before apparent hind limb licking or jumping was recorded. Each animal was tested three times with a 30 min break between the tests. For the treadmill experiment, the animals were placed individually in a housing apparatus equipped with a motorised treadmill with a 20% inclination (IITC, Los Angeles, CA, USA). An electrical shocking device was placed at the end of the treadmill. The treadmill was activated at a speed of 0.28 m/s ± 0.06 m/s for 6 min. The time elapsed before the shocking device was activated by the animals was recorded.

**IHC**

Cartilage specimens from humans and knee joints from mice were fixed with 4% paraformaldehyde for 48 hours, decalcified in 10% EDTA, embedded in paraffin, and sectioned at 5 μm. Sections were stained with 0.1% Safranin O and 0.001% fast green solution or Alcian blue solution (pH=2.5) to reveal the bone and cartilage. For IHC, the slides were incubated with sodium citrate antigen retrieval solution (Solarbio, Beijing, China) at 60°C overnight. Endogenous peroxidase activity was blocked by incubation with 3% hydrogen peroxide for 10 min. The slides were washed three times with PBS and blocked with 5% bovine serum albumin (BSA) in PBS for 1 h at room temperature. Slides were incubated with antibodies against MMP13, ADAMTS5, collagen II, aggrecan or TIMP3 (at 1:200 dilution, Abcam) at 4°C overnight. After washing three times with PBS, the slides were incubated with HRP-conjugated goat anti-rabbit secondary antibodies (ZSGB-Bio, Beijing, China) for 1 hour at room temperature and were then washed 3 times with PBS. The slides were developed with diaminobenzidine tetrahydrochloride (ZSGB-Bio, China) for three min at room temperature. Images of slides were acquired using a microscope (CX33TRF, Olympus, Tokyo, Japan). Three blinded scorers assessed the IHC-stained tissues according to the modified Pritzker OARSI score in Supplementary Table S1. The final scores are presented as the mean ± SEM values. Quantitative analysis was conducted in a blinded manner using Image-Pro Plus software.

**MicroCT analysis**

The knee joints from sacrificed animals were dissected from the distal femora to the proximal tibiae. The specimens were fixed with 4% paraformaldehyde for 48 h, washed, and stored in 70% ethanol. Then, the specimens were scanned using a high-resolution μCT (Skyscan 1072; Skyscan, Aartselaar, Belgium) using the following settings: X-ray voltage, 55 kV; current, 181 mA; acquisition time, 110 min; and resolution, 9 nm. After reconstruction, a sagittal plane of interest around the midline suture of the knee joint was chosen to assess osteophyte formation.

**Statistical analysis**

Statistical analyses were performed using SPSS 20 software (Abbott Laboratories, Chicago, IL, USA). Statistical significance was determined using unpaired Student’s t-tests unless indicated otherwise. Correlation studies were performed using Pearson’s correlation test, when appropriate. Results were considered statistically significant at P ≤ 0.05.
